# Noxious Iron–Calcium Connections in Neurodegeneration

**DOI:** 10.3389/fnins.2019.00048

**Published:** 2019-02-12

**Authors:** Marco Tulio Núñez, Cecilia Hidalgo

**Affiliations:** ^1^Iron and Neuroregeneration Laboratory, Department of Biology, Faculty of Sciences, Universidad de Chile, Santiago, Chile; ^2^Calcium Signaling Laboratory, Biomedical Research Institute, CEMC, Physiology and Biophysics Program, Institute of Biomedical Sciences, Faculty of Medicine, Universidad de Chile, Santiago, Chile; ^3^Department of Neuroscience, Faculty of Medicine, Universidad de Chile, Santiago, Chile

**Keywords:** neurodegenerative diseases, reactive oxygen species, mitochondria, HIF-1, Nrf-2, inflammation, ferroptosis

## Abstract

Iron and calcium share the common feature of being essential for normal neuronal function. Iron is required for mitochondrial function, synaptic plasticity, and the development of cognitive functions whereas cellular calcium signals mediate neurotransmitter exocytosis, axonal growth and synaptic plasticity, and control the expression of genes involved in learning and memory processes. Recent studies have revealed that cellular iron stimulates calcium signaling, leading to downstream activation of kinase cascades engaged in synaptic plasticity. The relationship between calcium and iron is Janus-faced, however. While under physiological conditions iron-mediated reactive oxygen species generation boosts normal calcium-dependent signaling pathways, excessive iron levels promote oxidative stress leading to the upsurge of unrestrained calcium signals that damage mitochondrial function, among other downstream targets. Similarly, increases in mitochondrial calcium to non-physiological levels result in mitochondrial dysfunction and a predicted loss of iron homeostasis. Hence, if uncontrolled, the iron/calcium self-feeding cycle becomes deleterious to neuronal function, leading eventually to neuronal death. Here, we review the multiple cell-damaging responses generated by the unregulated iron/calcium self-feeding cycle, such as excitotoxicity, free radical-mediated lipid peroxidation, and the oxidative modification of crucial components of iron and calcium homeostasis/signaling: the iron transporter DMT1, plasma membrane, and intracellular calcium channels and pumps. We discuss also how iron-induced dysregulation of mitochondrial calcium contributes to the generation of neurodegenerative conditions, including Alzheimer’s disease (AD) and Parkinson’s disease (PD).

## Introduction

Iron and calcium ions are both essential for maintaining normal brain function. Iron is required for oxidative phosphorylation, Krebs cycle, iron–sulfur cluster and heme synthesis, synaptic plasticity, and the development of cognitive functions (Oexle et al., 1999; Bolton et al., 2000; Hidalgo and Núñez, 2007;Tong and Rouault, 2007; Zorov et al., 2007; Huang et al., 2011; Muñoz et al., 2011; Murray-Kolb, 2013; Mena et al., 2015; Requejo-Aguilar and Bolanos, 2016), while increases in cytoplasmic calcium concentration known as calcium signals mediate the secretion of neurotransmitters, synaptic plasticity, and axonal growth (Berridge, 1998). Moreover, nuclear calcium signals command the expression of neuronal genes involved in learning and memory processes (Bading, 2013).

Recent studies have revealed that cellular iron stimulates neuronal calcium signaling, leading to downstream activation of kinase cascades engaged in synaptic plasticity, a neuronal response associated to memory and learning (Hidalgo and Núñez, 2007; Hidalgo et al., 2007; Muñoz et al., 2011). The relationship between calcium and iron is Janus-faced, however. Dysregulation of iron levels induces calcium dyshomeostasis and abnormal calcium signaling, whereas increased calcium levels enhance redox-active iron levels; neurodegenerative conditions exhibit both types of dysregulation. Thus, current evidence indicates that increased intracellular calcium and aberrant calcium signaling occur in neurodegenerative disorders that include Alzheimer’s disease (AD), Parkinson’s disease (PD), and amyotrophic lateral sclerosis (ALS) (reviewed in Pchitskaya et al., 2018). Similarly, disruption of neuronal iron homeostasis linked to iron accumulation is a common feature in a plethora of neurodegenerative diseases, which included and other parkinsonisms (Lee et al., 2013), AD (Bulk et al., 2018), Friedrich’s ataxia, pantothenate kinase-associated neurodegeneration (Swaiman, 1991), and other neuronal pathologies that entail brain iron accumulation (Wiethoff and Houlden, 2017).

Here, we will review the evidence that points to a rarely acknowledged relationship between iron homeostasis and calcium homeostasis and signaling, in which dysregulation of one of these processes results in dysregulation of the other, generating a vicious cycle that ends up in neuronal death. The multiple cell-damaging responses generated by the unregulated iron/calcium self-feeding cycle, such as excitotoxicity, free radical-mediated lipid peroxidation, and the oxidative modification of crucial components of iron and calcium homeostasis and signaling, including the iron transporter DMT1, plasma membrane and intracellular calcium channels and pumps will be presented. This review article concludes with a discussion of how dysregulation of calcium homeostasis and signaling contributes to the generation of neurodegenerative diseases with an iron accumulation component, focusing in particular on AD and PD.

## Iron Homeostasis in Neuronal Cells

Through the Fenton reaction, redox-active iron is a net producer of the hydroxyl radical, the most reactive radical species in nature (Davies, 2005). In a reductive environment, such as the intracellular milieu, through redox cycling redox-active iron promotes the production of hydroxyl radicals at the expense of O_2_ and GSH consumption (Núñez et al., 2012). Consequently, under physiological conditions, redox-active iron levels that conform the labile iron pool are very tightly regulated to a cytosolic concentration of 0.5–1.5 μM, which comprises <5% of total intracellular iron (Cabantchik, 2014). In mammals, cellular iron levels are maintained by transcriptional, translational, and vesicular flux mechanisms (Núñez, 2010). The iron regulatory protein (IRPs: IRP1 and IRP2)/iron-responsive element (IRE) is the best-characterized system that regulates at the translation level the expression of iron homeostasis proteins. These proteins include the transferrin receptor 1 (TfR1, involved in iron uptake), the divalent metal transporter 1 (DMT1, involved in iron uptake), ferroportin 1 (FPN1), involved in iron efflux from cells), and ferritin (involved in iron storage) (Wilkinson and Pantopoulos, 2014). The IRP/IRE is a plentiful-oriented system, since it is activated under low iron conditions to ensure an adequate supply of iron for cellular processes. Of note, the IRE–IRP regulatory system is not only regulated by cellular iron status but it is also regulated by reactive oxygen species (ROS), whereby cells elicit a defense mechanism against iron toxicity and iron-catalyzed oxidative stress (Ray et al., 2012).

The iron uptake protein DMT1 (SLC11A2) is a crucial component of cell iron homeostasis. *SLC11A2* transcription generates four alternatively spliced mRNAs that differ at their 5′-untranslated region (coding for the DMT1 isoforms 1A and 1B) and at its 3′-untranslated region (coding for isoforms +IRE and –IRE) (Garrick et al., 2006). Thus, the expression of the 1A and 1B isoforms of DMT1 is subjected to differential transcriptional regulation.

## The Crucial Relationship Between Iron and the Hypoxia-Inducible Transcription Factor (HIF)

At the systemic level, the hypoxia-inducible transcription factor (HIF) transcription factor family coordinates the cellular response to low oxygen levels by regulating the expression of a large array of target genes during hypoxia, which results in adaptive changes in the hematopoietic, cardiovascular, and respiratory systems (Smith et al., 2008; Mole et al., 2009). The HIF-1 is kept at basal levels by HIF prolyl hydroxylase domain (PHD) enzymes; prolyl-hydroxylation of HIF-1 via PHD signals for its degradation via the ubiquitin-proteasome system (Bruick and McKnight, 2001; Myllyharju, 2013; Yeh et al., 2017). The PHD enzymes are both oxygen- and iron-dependent; thus, hypoxia and iron chelation results in decreased PHD activity and increased HIF-1α activity (Hewitson et al., 2003; Nandal et al., 2011; Flagg et al., 2012).

In recent years, a series of iron chelating agents that exert neuroprotective effects have been developed (Núñez and Chana-Cuevas, 2018). In particular, M30, which is an 8-hydroxyquinoline-based iron chelator developed by the group of Moussa Youdim at Technion-Israel Institute of Technology (Weinreb et al., 2016), stabilizes HIF-1α, most probably by inactivating HIF-1α PHD. In the brain, HIF-1α stabilization by M30 leads to the expression of a broad number of neuroprotective-adaptive mechanisms and pro-survival signaling pathways (Kupershmidt et al., 2011). Real-time RT-PCR revealed that M30 differentially induces the expression of a variety of cellular components, including vascular endothelial growth factor, erythropoietin, enolase-1, TfR1, heme oxygenase-1, inducible nitric oxide synthase (iNOS), glucose transporter 1, brain-derived neurotrophic factor (BDNF), glial cell-derived neurotrophic factor, and the antioxidant enzymes catalase, superoxide dismutase-1, and glutathione peroxidase (Kupershmidt et al., 2009). Further reports have supported the role of iron chelators in inducing neuronal survival pathways (Mechlovich et al., 2014; Guo et al., 2015, 2016; Xiao et al., 2015). It follows that the capacity of iron chelators to induce HIF-1α-mediated neuroprotection adds to the recognized neuroprotective effects of iron chelators, through their ability to prevent hydroxyl radical production via the Fenton reaction. A tempering note comes from the report that treatment of human skin cells with the iron chelator *N*-(2-hydroxybenzyl)-L-serine (HBSer) does not induce HIF-1α activation, as opposed to desferrioxamine (DFO) and salicylaldehyde isonicotinoyl hydrazone (SIH) used as positive controls (Creighton-Gutteridge and Tyrrell, 2002). The authors conjectured that the lack of HIF-1α activation by HBSer might be related to its lower affinity for iron as compared to DFO and SIH. Of relevance to the theme of this review, however, is the fact that the transcription factor HIF-1α activates the expression of several genes associated with iron homeostasis (Lee and Andersen, 2006), which in non-excitable cells results in an increase in cellular iron content (Qian et al., 2011).

## Increased Reactive Oxygen/Nitrogen Species Generation Induces Iron Dyshomeostasis

A significant number of studies have shown that physiological levels of ROS and reactive nitrogen species (RNS) act as signaling molecules in a variety of biological responses (Sen, 2001; Ray et al., 2012; Asiimwe et al., 2016; Lourenco et al., 2017; Moldogazieva et al., 2018; Nemes et al., 2018). The brain is an organ highly susceptible to oxidative stress (Cobley et al., 2018). Hence, neuronal cells have to maintain physiological levels of ROS and RNS to avoid oxidative or nitrosative stress, which arises when excessive ROS/RNS production overcomes the cellular antioxidant systems, which by affecting the redox environment favors excitotoxicity. Henceforth, we will use the broad term oxidative stress to refer to both oxidative and nitrosative stress. Oxidative stress perturbs the function of many cellular processes; it causes glutathione deficiency (Wu et al., 2004) and promotes cell death by inducing mitochondrial depolarization, membrane lipid peroxidation, and DNA damage, among other harmful effects. To counteract the damaging effects of excess ROS/RNS (Duncan and Heales, 2005; Halliwell, 2006; Moncada and Bolaños, 2006), cells make use of enzymatic and non-enzymatic defense mechanisms, some of which engage calcium signals (Gordeeva et al., 2003). Depending on the extent and persistence of the cellular damage, oxidative stress may play a key role as a causative agent of neurodegenerative disease emergence and or progression.

Iron uptake into primary hippocampal neurons stimulates ROS generation (Muñoz et al., 2011), and decreases the redox potential established by the intracellular levels of oxidized and reduced glutathione in neuroblastoma cells (Núñez et al., 2004). The relationship between increased redox-active iron and increased oxidative stress is well accepted (Núñez et al., 2012). Similarly, in cellular models convincing evidence points to a link between increased oxidative tone and iron accumulation. Initial observations indicated that hydrogen peroxide activates IRP1 (Martins et al., 1995; Pantopoulos and Hentze, 1995; Hanson and Leibold, 1999; Caltagirone et al., 2001; Mueller et al., 2001), and that this activation results in increased cellular iron uptake mediated by increased translation of TfR1 and decreased ferritin synthesis (Caltagirone et al., 2001; Sureda et al., 2005). A subsequent study reported that, besides activation of IRP1, exposure of SH-SY5Y cells to H_2_O_2_ results in the degradation of the exo-transporter FPN1, which coupled to a decrease in ferritin translation leads to a time-dependent increase in the cellular labile iron pool (Dev et al., 2015), with the ensuing generation of oxidative damage through the production of hydroxyl radicals by the Fenton reaction. In animal models, evidence linking increased ROS as a cause, and iron homeostasis loss as an effect, is still lacking. An early report indicated that vitamin A deficiency results in increased hepcidin mRNA expression and increased iron spleen concentrations, accompanied by increased protein oxidation. These results suggest that vitamin A protects the liver against protein oxidation by maintaining iron homeostasis (Arruda et al., 2009). In a mouse model of experimentally induced hemolysis, excess heme increases intracellular ROS production, which signals for the induction of the unique iron exporter ferroportin, resulting in iron export from macrophages (Jeney et al., 2002; Marro et al., 2010). Of note, decreasing ROS levels by treatment with the antioxidant N-acetylcysteine prevents ferroportin induction and normalizes intracellular iron levels in a mice model of experimentally induced hemolysis (Tangudu et al., 2018). In a separate study using the APPswe/PS1ΔE9 AD mice model, administration for 3 months of antioxidants of plant origin decreased iron levels and malondialdehyde content and increased antioxidant defenses (Zhang et al., 2018). Overall, these results are consistent with the notion that oxidative stress dysregulates iron homeostasis both in cellular and animal models.

Nitric oxide also modifies IRP1 activity; it increases IRP1 binding to IRE and the increase IRE binding activity of IRP1 is coincident with inhibited ferritin synthesis and increased TfR1 mRNA levels (Phillips et al., 1996). Nitric oxide activates IRP1 directly by destabilizing the 4Fe-4S cluster of IRP1 (Drapier et al., 1993; Soum and Drapier, 2003), and may activate IRP1 indirectly by mobilizing intracellular Fe, thus decreasing the intracellular labile iron pool and activating IRP1 (Pantopoulos et al., 1996; Wardrop et al., 2000).

Altogether, these studies point to a robust association between increased ROS, nitric oxide, and increased levels of redox-active iron, which in turn mediate oxidative damage to lipids, protein, and nucleic acids. Yet, a recent study showed that in cultured macrophages, the labile iron pool apparently attenuates peroxynitrite-dependent damage (Damasceno et al., 2018). Whether this protective effect of the labile iron pool is translatable to other cell types and to neurons in particular, remains to be established.

## Iron Dyshomeostasis in Neurodegeneration

Increased iron levels and the associated ROS generation are important initiators and mediators of cell death (Dixon and Stockwell, 2014), whereas iron dyshomeostasis has a key role in neurodegeneration. Mutations of genes encoding proteins involved in iron homeostasis are associated with degeneration of central nervous system (CNS) cells (Ponka, 2004; Zecca et al., 2004), while oxidative stress induces cell injury by disrupting cellular iron balance (Carocci et al., 2018; Kerins and Ooi, 2018), thus leading to a vicious circle. Both AD and PD entail increased brain iron content and oxidative stress (Crichton et al., 2011; Ward et al., 2014; Tonnies and Trushina, 2017; Guo et al., 2018), with the implied threat of hydroxyl radical-induced neuronal damage. Of note, binding of Cu or Fe to Aβ peptides generates H_2_O_2_ (Huang et al., 1999), which may induce neuronal oxidative stress during AD progression (Cristovao et al., 2016). Moreover, iron overload increases neuronal amyloid-β production and enhances cognitive impairment in a transgenic mice model of AD (Becerril-Ortega et al., 2014) while accumulating evidence suggests that impaired iron homeostasis is an early event in AD progression (Peters et al., 2015).

Parkinson’s disease is a progressive neurodegenerative disease characterized by rigidity, tremor, and slowness of movement as well as by a wide range of debilitating non-motor symptoms. A key feature of PD is the selective loss of the dopaminergic neurons of the substantia nigra; ample evidence shows iron accumulation in these neurons (Jellinger, 1999; Zecca et al., 2004; Berg and Youdim, 2006; Muñoz et al., 2016; An et al., 2018). The observation that pharmacological agents with iron chelation capacity prevent neuronal death in AD or PD experimental models (recently reviewed in Núñez and Chana-Cuevas, 2018) highlights the pivotal role of iron as a mediator of neuronal death in AD and PD.

In addition, spinal neurons from ALS patients display increased iron and calcium levels (Kasarskis et al., 1995). The success of iron chelation therapy in ALS mouse models sustains the central role of iron in ALS pathogenesis (Jeong et al., 2009; Kupershmidt et al., 2009). Alterations of proteins involved in iron metabolism, inhibition of anterograde axonal transport leading to iron accumulation in ventral motor neurons, and increased mitochondrial iron load in neurons and glia have been proposed as pathogenic mechanisms to explain the abnormal iron accumulation displayed by the neurons and glia of ALS mice (Jeong et al., 2009).

## Iron Dyshomeostasis in Inflammation

Inflammation, a condition often found in neurodegenerative environments, is yet another factor that induces iron accumulation (reviewed in Urrutia et al., 2014). Both the p50 and the p65 subunits of the transcription factor NF-κB bind to a NFκB-responsive element present in the 5′ untranslated region of the DMT1-1B promoter, inducing the expression of the 1B isoforms of DMT1 (Paradkar and Roth, 2006a,b). The transcription factor NF-κB is often activated by inflammatory signals (Rothwell and Luheshi, 2000; Hanke and Kielian, 2011; Shih et al., 2015). Accordingly, the possible association between inflammation and iron accumulation has been recently explored (Pelizzoni et al., 2013; Urrutia et al., 2013, 2014; McCarthy et al., 2018). To this purpose, the effects of lipopolysaccharide (LPS) and the pro-inflammatory cytokines TNF-α and IL-6 on mRNA and protein levels of DMT1, FPN1, and hepcidin were investigated in astrocytes, microglia, and neurons isolated from rat brain. A causal association between inflammation and iron accumulation was reported, since in addition to increasing neuronal iron content, treatment with LPS, TNF-α, or IL-6 increases DMT1 expression and protein levels in neurons (Urrutia et al., 2013). Similar results were reported in studies of ventral mesencephalic neurons in primary culture, in which treatment with TNF-α or IL-1β induces an increment in DMT1 and TfR1 protein levels, together with a reduction of FPN1 levels (Wang et al., 2013). Considering that NFκB activation is downstream of the TNF-α, IL-1, and LPS signaling pathways, these results are consistent with a circuit in which inflammatory stimuli induce DMT1 expression and iron accumulation via NFκB activation and hepcidin expression.

An additional link between inflammation and increased oxidative stress resides in the observation that inflammatory stimuli activate the NADPH oxidase (NOX) enzyme, the main regulated source of cellular ROS generation (Green et al., 2001; Urrutia et al., 2014; Sorce et al., 2017). In particular, pro-inflammatory cytokines activate the phagocytic NOX2 enzyme, which is highly expressed in microglia (Sorce and Krause, 2009; Lim et al., 2013; Barrett et al., 2017). In turn, NOX2 activation generates an oxidative extracellular environment that increases ROS levels in neighboring neurons (Schiavone et al., 2009; Gao et al., 2012; Surace and Block, 2012; Zhang et al., 2014).

Of note, NOX activation has also been reported in experimental models of PD and AD. In particular treatment with MPTP (1-methyl-4-phenyl-1,2,3,6-tetrahydropyridine), a prodrug to the Parkinsonian neurotoxin 1-methyl-4-phenylpyridinium (MPP+), results in increased synthesis of the pro-inflammatory cytokine IL-1β, and increased membrane translocation of the cytosolic NOX sub-unit p67^phox^, which is prevented by minocycline, a tetracycline derivative that exerts multiple anti-inflammatory effects (Wu et al., 2002). In addition, iron supplementation to midbrain neuron-glia cell cultures causes the selective death of dopaminergic neurons. This death is mediated by microglial NOX2 since cells derived from NOX2-/- mice do not present iron-induced neuronal death (Zhang et al., 2014). Similarly, brain tissue from AD patients reveals an elevation in NOX activity coupled to membrane translocation of cytosolic NOX2 subunits and increased NOX1 and NOX3 mRNA transcripts (Shimohama et al., 2000; Ansari and Scheff, 2011). Significantly, the authors found an inverse correlation between postmortem NOX activity and ante-mortem cognitive status (Ansari and Scheff, 2011). Altogether, these results indicate that inflammatory stimuli generate a neuronal phenotype of increased iron influx and increased NOX-produced ROS, with the consequent risk of oxidative damage observed in neurodegenerative diseases.

## Role of Iron in Excitotoxicity

Earlier reports indicate that iron plays a role in excitotoxicity, a death process mediated by high levels of intracellular calcium caused by excessive activity of excitatory neurotransmitters. Seminal observations by Cheah and colaborators described a signaling cascade in which stimulation of *N*-methyl-D-aspartate (NMDA) receptors activates the small GTPase Dexras1 via S-nitrosylation, mediated by activation of neuronal nitric oxide synthase (nNOS). Activated Dexras binds to the peripheral benzodiazepine receptor-associated protein PAP7, which in turn binds to the iron transporter DMT1 (Cheah et al., 2006). Most importantly, treatment with neurotoxic concentrations of NMDA elicits a major increase in iron uptake, resulting in three- to fivefold increase of hydroxyl radical levels and the death of more than 90% of cells, whereas co-treatment with NMDA and a cell-permeant iron chelator largely blocked NMDA-induced cell death. Overall, these results indicate that glutamate-NMDA excitotoxicity elicits DMT1 activation and iron-mediated cell death (Cheah et al., 2006). Although the role of calcium was not explored, the authors hypothesized initial calcium entrance through the NMDA receptor may activate nNOS, thus initiating this neurodegenerative process. As discussed below, crosstalk between NMDA receptor-mediated calcium influx and iron is likely to have a relevant role in neurodegenerative diseases such as AD and PD.

## Neuronal Calcium Homeostasis and Signaling

Calcium is a universal second messenger that regulates numerous cellular processes over a wide temporal range (Berridge et al., 2003). In neurons, the free calcium concentration at rest lies in the 70–100 nM range (Clapham, 2007), whereas extracellular calcium concentration is ∼1.5 mM. This large concentration gradient, combined with resting potential values of 70–90 mV (negative inside), generate a significant electrochemical gradient that favors calcium entry. Calcium homeostasis and signaling are vital for cellular function and survival; thus, neuronal cells possess powerful systems to maintain calcium homeostatic and signaling systems (Berridge, 1998; Brini et al., 2014), which regulate the neuronal processes underlying synaptic plasticity and complex brain cognitive functions, such as information processing, learning, and memory. Calcium is stored also in intracellular organelles such as the endoplasmic reticulum (ER), where its free concentration lies in the 0.5 mM range. The main systems in charge of maintaining calcium homeostasis are two plasma membrane calcium (PMCA) transporters, the PMCA pump and the sodium/calcium exchanger (NCX), plus the sarco/ER Ca^2+^-ATPase (SERCA) pump that maintains the steep concentration gradient between the ER and the cytoplasm (Brini et al., 2014). These transporters utilize different mechanisms and display a range of calcium affinities, transport rates, and capacities to ensure the proper managing of the variety of intracellular increases in free calcium concentration, known as calcium signals, generated by the diverse stimuli neuronal cells receive.

Neuronal activity generates calcium signals that via sequential activation of calcium-dependent signaling cascades and transcription factors promote the expression of genes underlying dendritic development, synaptic plasticity, and neuronal survival (Brini et al., 2014). In response to neuronal stimulation, calcium influx through plasma membrane pathways, including agonist-operated, store-operated, or voltage-gated calcium channels (VGCCs), generates neuronal calcium signals. In addition, the release of calcium from intracellular stores such as the ER also contributes to neuronal calcium signal generation (Brini et al., 2014). All neuronal compartments crucial for neurotransmission have the two types of calcium release channels: the inositol 1,4,5-trisphosphate (IP_3_) receptor (IP_3_R) and the ryanodine receptor (RyR) channels (Berridge, 1998). These large channels have complex gating and regulation and mediate a cellular process known as calcium-induced calcium release (CICR), whereby calcium promotes its own release from intracellular calcium stores (Berridge, 1998). This CICR mechanism likely contributes to propagate calcium signals that reach distant targets such as the nucleus (Berridge, 1998), where calcium promotes short or long-lasting changes in neuronal function and structure (Bading, 2013).

## ROS Levels Influence Calcium Homeostasis and Signaling

Significant evidence gathered in the last years indicates that increased cellular ROS levels modify the function of key proteins engaged in calcium homeostasis and signaling (Gordeeva et al., 2003; Hidalgo and Donoso, 2008; Gorlach et al., 2015). Redox-dependent regulation of components that maintain neuronal Ca^2+^ homeostasis may influence the direction and/or the efficiency of Ca^2+^-signaling pathways. Interactions among ROS and calcium-dependent signaling pathways are bidirectional; while ROS regulate cellular calcium signaling, calcium signals are essential for ROS production because a number of ROS-generating and antioxidant systems of living cells are calcium-dependent (Gordeeva et al., 2003).

Reversible modifications of proteins via ROS-promoted oxidation of their amino acids modify the properties of neuronal proteins engaged in signal transduction, such as protein kinases, protein phosphatases, and transcription factors (Wang et al., 2012). An increase in cellular ROS levels results in the inhibition of NMDA receptor function and in decreased activity of both the PMCA and the SERCA calcium pumps, while it enhances the activity of IP_3_R and RyR calcium channels (Hidalgo and Donoso, 2008; Joseph et al., 2018). Consequently, ROS generation in neuronal cells increases cellular calcium levels by promoting calcium release mediated by IP_3_R and RyR channels and by inhibiting both the PMCA and the SERCA pumps: the ROS-mediated increase in calcium levels promotes changes in several calcium-dependent neuronal pathways (Yermolaieva et al., 2000; Riquelme et al., 2011). Depending on the magnitude of the calcium increase, activation of kinases or phosphatases implicated in synaptic plasticity process will occur (reviewed in Hidalgo and Arias-Cavieres, 2016). In addition, activation of the large-conductance Ca^2+^-activated K^+^ (BKCa) by oxidation of SH cysteine residues, by decreasing neuronal excitability reduces the vulnerability of hippocampal neurons to hypoxia (Hepp et al., 2005).

## Defective Calcium Homeostasis and Signaling in Neurodegeneration

A link between excessive intracellular calcium accumulation and neuronal death is by now firmly established (Demuro et al., 2010; Stefani et al., 2012; Fukui, 2016). Neuronal cells are particularly sensitive to calcium-mediated cytotoxic damage and the failure of neurons to maintain Ca^2+^ homeostasis is a common feature of aged-linked neurodegenerative pathologies (Berrocal et al., 2015; Brini et al., 2017; Pchitskaya et al., 2018; Strehler and Thayer, 2018). Calcium dyshomeostasis (Pchitskaya et al., 2018) and altered neuronal calcium signaling (Chan et al., 2009; Overk et al., 2015) have been reported in a number of disease conditions, including AD, PD, and ALS (for recent reviews, see Franco-Iborra et al., 2018; Popugaeva et al., 2018; Post et al., 2018; Sirabella et al., 2018). Likewise, defective calcium signaling in glia and autophagy-related pathways has been implicated in neurodegenerative disease (Mustaly-Kalimi et al., 2018). Accruing evidence implicates decreased levels and functional decline of PMCA isoforms in neurodegeneration (Hajieva et al., 2018; Strehler and Thayer, 2018). In addition, in many pathological conditions, disruptions of ER Ca^2+^ signaling disrupt neuronal function and promote neuronal cell death (Okubo et al., 2018). To date, however, the detailed mechanisms of how neuronal Ca^2+^ homeostasis and signaling are perturbed in neurodegenerative diseases are not well understood.

The most common neurodegenerative disorder and the leading cause of dementia in the elderly is AD, whereas PD is the most prevalent age-associated movement disorder that entails early and selective degeneration of dopaminergic neurons. The pathological hallmarks of AD are neuronal loss and the presence of amyloid plaques and neurofibrillary tangles in the brain of affected individuals. Yet, before the emergence of plaques and/or neurofibrillary tangles other defects, including Ca^2+^ and iron dyshomeostasis, mitochondrial dysfunction, increased contacts between the ER and the mitochondria, neuro-inflammation, and alterations in lipid metabolism have been reported (Mattson, 2010; Area-Gomez and Schon, 2017; Lane et al., 2018). How these early defects relate to the emergence and progression of AD remains to be established. Of note, a recent report by the Alzheimer’s Association Calcium Hypothesis Workgroup [AACHW] (2017) analyzed how defective calcium signaling may be an early upstream event in AD progression, and how alterations of the many cellular components that control calcium hemostasis and signaling leads to the decline in the function of more vulnerable neuronal cells. In particular, these more vulnerable neuronal cells present early synaptic dysfunction, upregulation of some calcium signaling related genes and significant decreases in the expression of genes related to synaptic neurotransmission, neurotrophic factor activity, mitochondrial metabolism, and energy production, among others (Alzheimer’s Association Calcium Hypothesis Workgroup [AACHW], 2017).

In PD, dopaminergic neurons of the substantia nigra are especially vulnerable to Ca^2+^ dyshomeostasis, because their autonomous pace-making activity, through which these neurons generate action potentials in the absence of synaptic input (Guzman et al., 2009). This particular property of dopaminergic neurons imposes a high pressure on adequate handling of activity-generated Ca^2+^ signals, presumably mediated by the significant calcium influx through plasma membrane L-type calcium channels that markedly surpasses the calcium influx displayed by other neurons (Surmeier et al., 2010).

Dopaminergic neurons are highly vulnerable to oxidants and mitochondrial inhibition compared to other neuronal types (Sherer et al., 2003; Halliwell, 2006; Burbulla et al., 2017). Thus, brain permeant toxins such as rotenone, an inhibitor of NADH dehydrogenase, exclusively affect dopaminergic neurons (Sherer et al., 2003; Cannon et al., 2009). This special vulnerability is likely to produce the mitochondrial Ca^2+^ imbalance that occurs in PD (Ludtmann and Abramov, 2018). As discussed below, iron-induced ROS increases result in calcium signal generation, which may contribute to the basal calcium burden of dopaminergic neurons.

Several studies have reported defective Ca^2+^ signaling in other neurodegenerative conditions such as ALS (Siklos et al., 1998; Grosskreutz et al., 2010; Jaiswal, 2013; Muhling et al., 2014) and multiple sclerosis (MS; Gonsette, 2008). The death of motor neuron is a characteristic feature of ALS. Excitotoxicity, which involves glutamate and calcium overload, or cell death caused by high levels of intracellular calcium caused by excessive activity of excitatory neurotransmitters, may contribute to ALS pathology. Calcium dysregulation is also present in MS, where the associated disability results from neuronal and axonal loss initiated by microglia activation and mediated by oxidative stress, excitotoxicity, calcium dysregulation, and mitochondrial dysfunction, leading to proteolytic enzyme production and apoptosis (Gonsette, 2008).

Additionally, calcium dysregulation has been associated with neuroinflammation, a CNS response that comprises biochemical and cellular responses to injury, infection, or neurodegenerative diseases (DiSabato et al., 2016). Neuroinflammation markers increase in some neurodegenerative conditions; microglia, the innate CNS immune cells, play key roles as mediators of these neuroinflammatory responses. Of note, Ca^2+^ and neuroinflammatory signaling mechanisms exhibit extensive crosstalk and bidirectional interactions. As an example, chronic brain inflammation leads to a feedback reduction in NMDA receptors caused by excess synaptic glutamate activity during microglial activation (Rosi et al., 2004).

Despite the abundant literature that points to defective calcium homeostasis and signaling in neurodegeneration, scarce information is available in the literature regarding the protective effects of calcium modulators in preventing these defects. Yet, all recent reports on this subject highlight the fact that this is an urgent task. The non-competitive NMDA receptor antagonist Memantine, which reduces excitoxicity by inhibiting neuronal calcium entry (Bezprozvanny and Mattson, 2008), has been the most common drug for treating moderate to severe AD; nevertheless, Memantine provides only limited benefits for AD patients. A recent report showed that immunotherapy with a murine analog of the anti-Aβ antibody Aducanumab restores calcium homeostasis in a transgenic AD mice model (Kastanenka et al., 2016), whereas a recent review addresses the evidence linking neurodegeneration to RyR dysfunction and describes novel therapeutic approaches to target abnormal RyR function (Kushnir et al., 2018). There are no reports, however, on the efficacy of these strategies for treating AD patients.

## The HIF-1–Calcium Relationship

In SH-SY5Y neuroblastoma cells, chelation of calcium by 1,2-bis(2-aminophenoxy)ethane-N,N,N’,N’-tetraacetic acid (BAPTA) induces HIF-1α protein accumulation and nuclear localization, whereas the cytosolic calcium elevation produced by inhibition of the SERCA pump attenuates these effects (Berchner-Pfannschmidt et al., 2004). Further experiments indicated that calcium chelation attenuates the interaction of HIF-1α with the von-Hippel-Lindau protein, thus decreasing the proteasomal degradation of HIF-1α (Berchner-Pfannschmidt et al., 2004). Control experiments indicated that BAPTA did not complex intracellular iron ions, thus the above effects were caused by calcium chelation and not by stimulation of iron-activated PHD enzymes (Berchner-Pfannschmidt et al., 2004). Subsequent studies showed that intermittent hypoxia causes HIF-1α accumulation through a mechanism that involves increased cell calcium and increased generation of ROS by NOX, which induces a complex signaling pathway that results in increased HIF-1α synthesis and decreased hydroxylase-dependent HIF-1α degradation (Yuan et al., 2008).

Although brain levels of HIF-1α are considered neuroprotective (Kietzmann et al., 2001; Siddiq et al., 2005; Guo et al., 2016; Lee et al., 2017; Merelli et al., 2018), under certain conditions, HIF-1α acts as a pro-apoptotic factor (Piret et al., 2002; Zhang et al., 2007; Merelli et al., 2018). For example, the anesthetic isoflurane induces apoptotic neurodegeneration in a process mediated by HIF-1α, since knockdown of HIF-1α expression attenuates isoflurane-induced neurotoxicity (Jiang et al., 2012). In addition, isoflurane induces a significant elevation of cytoplasmic calcium levels in cultured neurons. Based on previous observations, showing that calcium mediates HIF-1α expression (Yuan et al., 2005; Riganti et al., 2009), the authors hypothesized that the high calcium levels induced by isofluorane promote HIF-1α-mediated apoptotic neurodegeneration. Studies discerning the calcium concentrations and other cell physiological condition, which elicit each of the above responses, should shed light on these apparent contradictory responses.

Overall, the emerging picture from the above results is consistent with the idea that low calcium concentrations stabilize HIF-1α protein levels by decreasing its proteasomal degradation. The effect of calcium concentrations above homeostatic levels is not clear and may depend on cell type: high calcium levels may induce a protective response consisting in increased HIF-1α synthesis coupled to decreased degradation or may mediate HIF-1α degradation.

## Iron Modifies Neuronal Calcium Homeostasis and Calcium Signaling Pathways

Iron overload increases intracellular calcium levels, which in turn activate calcineurin (Lee et al., 2016), a calcium-dependent phosphatase which has a key role in synaptic plasticity and memory processes (Baumgartel and Mansuy, 2012). Increases in cytoplasmic calcium levels via the NMDA receptor stimulate the neuronal NOS enzyme (Ghosh and Salerno, 2003) and generate superoxide anion in hippocampal neurons (Brennan et al., 2009), while in cortical neurons, this calcium increase induces a signaling cascade that enhances superoxide generation through NOX activation (Girouard et al., 2009).

Both synaptic plasticity and memory processes require activity-dependent hippocampal ROS generation (Bindokas et al., 1996; Kahlert et al., 2005; Kishida and Klann, 2007). In primary hippocampal neurons, the ROS increase induced by iron promotes calcium release from the ER mediated by redox-sensitive RyR channels; the resulting increase in intracellular calcium levels link NMDA receptor stimulation to ERK1/2 activation and nuclear translocation (Muñoz, 2012). Since several reports indicate that CREB-dependent transcription of synaptic plasticity related genes entails ERK1/2-mediated long-term CREB phosphorylation (Wu et al., 2001; Lynch, 2004; Thomas and Huganir, 2004). Accordingly, these findings indicate that iron-induced, RyR-mediated, calcium release contributes to this activity-dependent neuronal response.

In summary, the current evidence suggests that crosstalk between ROS and calcium plays an essential role in many pathophysiological conditions including neurodegenerative diseases such as PD and AD. Here we complement this idea by adding the component of iron overload, and the ensuing oxidative stress, as a significant factor in promoting calcium dysregulation in neurodegeneration.

## The Synergism Between Iron and Calcium in Lipid Peroxidation

A link between iron-mediated lipid peroxidation and calcium dyshomeostasis was reported more than 30 years ago. Peroxidation of rat brain synaptosomes by Fe^2+^ and H_2_O_2_ generates rapid and large uptake of Ca^2+^ by isolated synaptosomes; Fe^2+^ also enhances Ca^2+^ uptake by spinal cord neurons in culture, an effect that is coupled to lipid peroxidation and the release of arachidonic acid from cells, in a process prevented by the iron chelator DFO (Braughler et al., 1985). The authors concluded that Fe^2+^ and Ca^2+^ act synergistically to produce lipid peroxidation and damage to neuronal membranes (Braughler et al., 1985). A further report proposed that this is not a direct effect of calcium but the result of independently initiated processes of lipid peroxidation and Ca^2+^ translocation, which interact subsequently in a synergistic manner (Bors et al., 1990).

The relationship between iron-induced lipid peroxidation and dysregulation of Ca^2+^-ATPase is long-dated (Pelizzoni et al., 2008; Mata and Sepulveda, 2010; Zaidi, 2010). Plasma membrane lipid peroxidation initiated by the Fenton reaction results in increased calcium content, derived from the inhibition of the plasma membrane and ER Ca^2+^-ATPases (Pereira et al., 1996). This inhibitory effect is shared by other radical species, in particular by peroxynitrite (Zaidi and Michaelis, 1999). Oxidants also inhibit SERCA activity while promoting calcium release from the ER (Gordeeva et al., 2003; Hidalgo and Donoso, 2008). Arguably, the observed upsurge in intracellular Ca^+2^ induced by iron-mediated lipid peroxidation could be compounded by an oxidant-induced loss of PMCA and SERCA activity, which combined with ROS-mediated activation of IP_3_R/RyR channels would result in further and deleterious increases in the intracellular Ca^2+^ concentration.

## Iron, Calcium, and HIF-1 Activity

**FIGURE 1:**
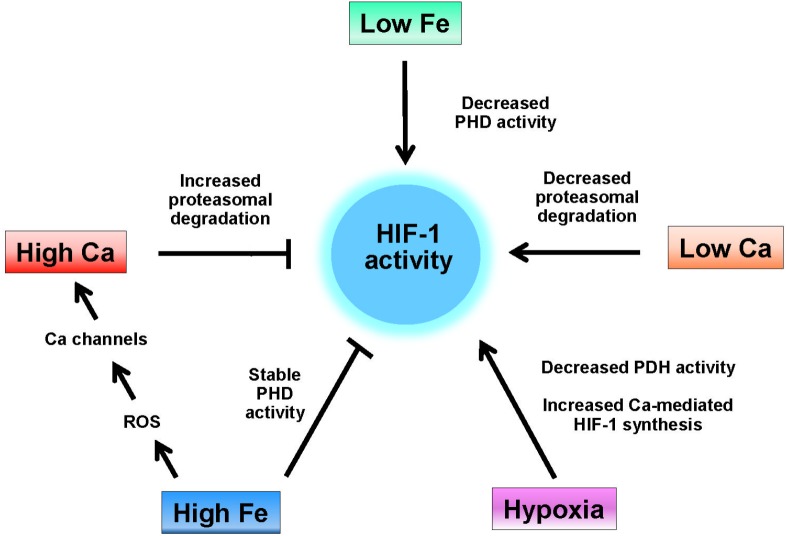
Regulation of HIF-1 activity by iron and calcium. HIF-1 activity is induced by hypoxia, which decreases the activity of HIF-1 hydrolases (PHD), by low iron (e.g., iron chelation), which also decreases the activity of HIF-1 hydrolases, and by low intracellular calcium, which decreases the interaction of HIF-1α with the von-Hippel-Lindau protein (see text). High intracellular calcium levels increase the rate of HIF-1 proteasomal degradation while high levels of iron both maintain the activity of HIF-1 hydrolases and, through enhanced ROS production increase the cellular levels of calcium by activation of RyR, IP_3_R, and L-type calcium channels.

Considering the overlapping effects of iron and calcium on HIF-1 activity detailed above, a Fe/Ca bipartisan regulation of HIF-1 can be hypothesized (Figure 1). In neurodegenerative diseases with an iron accumulation component, decreased HIF-1 activity is predicted by a mechanism that involves the maintenance of maximal HIF-1 hydrolase activities and the increase of intracellular calcium mediated by dysregulation of redox-sensitive calcium homeostatic and signaling pathways. In neurons, decreased HIF-1 activity results in decreased neuroprotective, neuroregenerative, and antioxidant responses associated to HIF-1 activity (see above).

**FIGURE 2:**
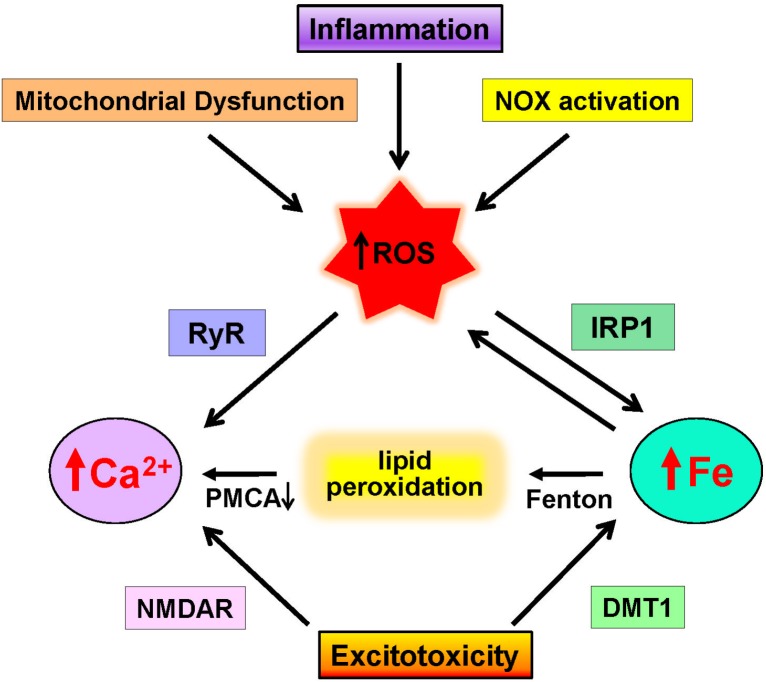
Neuronal pathways that increase calcium and iron levels. Increases in ROS due to mitochondrial dysfunction, inflammation, and NOX activation promote excessive ROS generation that promotes calcium release via RyR channels and increases redox-active iron by IRP1 activation. Through the Fenton reaction, iron induces lipid peroxidation that inhibits the PMCA calcium pump. In addition, excitotoxic conditions over-activate NMDA receptors (NMDAR), which results in increased cytoplasmic calcium levels and increases iron levels via DMT1 activation.

## Calcium-Induced Iron Dyshomeostasis: Possible Role in Neurodegeneration

Excessive intracellular calcium levels, such as those associated with neurodegenerative conditions, are likely to produce high H_2_O_2_ levels via NOX2 and to enhance mitochondrial calcium uptake and ROS production. These two ROS sources, NOX2, and mitochondria should affect jointly iron homeostasis by increasing the cellular labile iron pool, as occurs in SH-SY5Y cells exposed to H_2_O_2_ (Dev et al., 2015). These findings raise the possibility that conditions that increase postsynaptic calcium levels following NMDA receptor activation, which promote H_2_O_2_ generation in neocortical neurons via a cascade engaging the NOS and NOX2 enzymes (Girouard et al., 2009), may lead to an upsurge of the labile iron pool.

The role of voltage-gated Ca^2+^ channels as mediators of iron transport into neuronal cells is well established (Gaasch et al., 2007; Lockman et al., 2012). Another link between iron and calcium during neurodegeneration was provided by the observation that the hippocampal and nigral neuron loss induced by intra-cerebroventricular FeCl_3_ injection in rats is largely prevented by pre-treatment with nicardipine, a blocker of L-type VGCCs (Bostanci and Bagirici, 2013). The authors concluded that these channels mediate the neurotoxic effects of extracellular iron by inducing either calcium and/or iron uptake. The question remains as to whether the protective effect of nicardipine is due to decreased iron influx, decreased calcium influx, or to a calcium-mediated process leading to neurodegeneration downstream of iron-induced oxidative stress. Other studies have also provided evidence of iron influx through NMDA receptors or voltage-operated calcium channels in primary hippocampal neurons (Pelizzoni et al., 2011) and the dopaminergic MES23.5 cell line (Wang et al., 2017).

## The Mitochondrion Melting POT: Calcium, Iron, and Apoptotic Cell Death

Mitochondria, by taking up and releasing calcium, act as important regulators of cellular calcium levels. The large mitochondrial membrane potential difference (negative inside) provides the driving force for calcium entry through the mitochondrial calcium uniporter (MCU), a component of the inner mitochondrial membrane; in physiological conditions, calcium antiporters rapidly extrude calcium from the mitochondria and restore the basal calcium levels (Giorgi et al., 2018). Mitochondrial calcium concentration modulates ATP production and mitochondrial metabolism, whereas mitochondrial calcium overload induced by deregulated intracellular calcium levels promotes apoptotic cell death, necrosis, and autophagy (Giorgi et al., 2012; Rimessi et al., 2013; Marchi et al., 2018). In pathological conditions, the MCU has been implicated in excitotoxicity, iron overload, inflammation, and oxidative stress-induced mitochondrial dysfunction and cell death (Liao et al., 2017). The ROS generated by mitochondrial enzymes or the electron transport chain presumably regulate diverse cellular physiological pathways, whereas dysregulated ROS generation may contribute to the development of human diseases (Angelova and Abramov, 2017).

Neurodegenerative disorders such as AD, PD, and ALS display dysregulation of calcium homeostasis and mitochondrial dysfunction; in addition, excitotoxicity leads to mitochondrial calcium overload and promotes necrotic or apoptotic-like excitotoxic cell death (Hansson Petersen et al., 2008; Rosales-Corral et al., 2012; Bose and Beal, 2016; Gibson and Thakkar, 2017; Giorgi et al., 2018). An increase in mitochondrial calcium is of particular importance in dopaminergic neurons, which are constantly exposed to calcium influx (Surmeier et al., 2011). The interaction between calcium overload and excessive ROS production (Chinopoulos and Adam-Vizi, 2006) causes further damage to the electron transport chain and promotes oxidative stress, which induces the oxidation of mitochondrial lipids, proteins, and DNA leading to cytotoxicity (Muravchick and Levy, 2006). The vicious cycle between calcium overload and oxidative stress favors the sustained opening of the mitochondrial permeability transition pore (mPTP), leading to the collapse of the mitochondrial membrane potential and mitochondrial swelling, which in turn promote the apoptotic response (Di Lisa and Bernardi, 2009). Opening of the mPTP has a central role in the pathogenesis of neurodegenerative disorders (Vila and Przedborski, 2003; Perier et al., 2005).

Excessive iron levels promote oxidative stress leading to the upsurge of unrestrained calcium signals that damage mitochondrial function. Iron accumulation in the cytoplasm induces excessive iron influx into mitochondria through mitoferrin1/2 (Paradkar et al., 2009), which causes mitochondrial dysfunction by increased ROS production leading to oxidative damage. Hence, if uncontrolled, this calcium/iron self-feeding cycle becomes deleterious to mitochondrial and neuronal function, leading eventually to neuronal death. In this regard, it is of note that, by preserving mitochondrial calcium homeostasis, the iron-binding protein lactoferrin protects vulnerable dopamine neurons from degeneration (Rousseau et al., 2013).

In primary hippocampal neurons, iron-induced stimulation of redox-sensitive RyR-mediated calcium release causes significant mitochondrial fragmentation (Sanmartin et al., 2014). Iron overload induces oxidative stress in the ER earlier than in the mitochondria, thereby increasing ER stress and calcium levels, and consequently causing mitochondrial fragmentation and neuronal cell death (Lee et al., 2016). Mitochondrial fragmentation presumably contributes to the impairment of neuronal function produced by iron overload, since inhibition of mitochondrial division protects neuronal cells from excitotoxicity (Ruiz et al., 2018). Moreover, iron overload of HT-22 hippocampal neuron cells increases intracellular calcium levels, causes mitochondrial fragmentation via dephosphorylation of Drp1, and increases apoptotic neuronal death; of note, calcium chelation and inhibition of calcineurin prevented mitochondrial fragmentation and cell death (Lee et al., 2016). Based on these findings, the authors suggested that calcium-mediated calcineurin signaling, by regulating mitochondrial dynamics, has a key role in iron-induced neurotoxicity.

In summary, mitochondrial calcium overload combined with excessive iron-mediated ROS generation and mPTP opening compose a cell death-inducing sequence implicated in neuronal dysfunction and neurodegeneration.

## Oxytosis/Ferroptosis a Possible Meeting Point for Calcium and Iron-Induced Cell Death

Oxytosis was first described as a glutamate-induced, calcium-dependent form of cell death (Murphy et al., 1988). Initial studies on the molecular mechanisms of oxytosis pointed to a decrease in intracellular glutathione levels, enhanced mitochondrial ROS production, lipid peroxidation, and massive calcium influx as causes of death (Miyamoto et al., 1989). The mechanistic link between glutamate exposure and glutathione depletion was the inhibition of the cysteine/glutamate antiporter xc- by glutamate, which deprived cells of cystine, a precursor in glutamate synthesis (Murphy et al., 1989). As glutathione levels decrease, ROS levels increase exponentially (Tan et al., 1998), which gives rise to the activation of signaling pathways that culminate in large influx of calcium and cell death (Maher et al., 2018). Thus, the increased calcium influx is an essential mediator of the cell death program, as cells do not die in calcium-free medium.

Ferroptosis is a form of regulated cell death that occurs because of lethal lipid peroxidation. Ferroptotic cell death is blocked by iron chelators, lipophilic antioxidants, and by agents that block propagation of lipid peroxidation (Stockwell et al., 2017; Fricker et al., 2018). Ferroptosis is induced by inhibition of the cystine-glutamate antiporter xc- or by inhibition of glutathione peroxidase 4 (Gpx4). Induction of ferroptosis by erastin (an inhibitor of the antiporter xc-) results in cell death, which is morphologically characterized by mitochondrial shrinkage, with no overt signs of apoptosis or necrosis (Dixon et al., 2012). Ferroptotic death derives from depletion of cell glutathione, increased oxidative stress, and iron-mediated lipid peroxidation to lethal levels (Stockwell et al., 2017). Inhibition of Gpx4 also causes ferroptosis (Yang et al., 2014). Gpx4 is a lipid peroxidase that directly reduces phospholipid hydroperoxides at the expense of reduced glutathione. In mice, conditional deletion in forebrain neurons of Gpx4 resulted in hippocampal neurodegeneration and behavior dysfunction, associated with markers of ferroptosis such as elevated lipid peroxidation, ERK1/2 activation, and neuroinflammation (Hambright et al., 2017). Given the large similarity in their cell death mechanisms, the current view is that oxytosis and ferroptosis are similar forms of cell death (Fricker et al., 2018; Lewerenz et al., 2018; Maher et al., 2018).

Although a lethal influx of calcium is an intrinsic feature of oxytosis, the participation of calcium in ferroptosis is still poorly defined. Initial work by Dixon et al. (2012) revealed that calcium chelators do not inhibit erastin-induced HT-1080 cell death. Nevertheless, as mentioned above, an increase in cellular ROS levels enhances the activity of IP_3_R and RyR calcium release channels and decreases the activity of both the PMCA and the SERCA calcium pumps. These effects increase cellular calcium levels by promoting calcium release from the ER while inhibiting its removal by the calcium pumps.

Lipid peroxidation-derived modification of some calcium channels could also be intermediates in ferroptotic death (Maher et al., 2018). In particular, 4-hydroxynonenal, a product of hydroxyl radical-mediated lipid peroxidation, induces the opening of NMDA receptor and VGCCs (Lu et al., 2002). Indeed, the store-operated calcium entry (SOCE) system, which activates calcium influx channels in the plasma membrane upon the release of intracellular calcium from the ER participates in the neuronal death induced by MPP+, a Parkinsonian toxin that inhibits mitochondria electron transport chain complex I. Inhibition of the SOCE response decreased apoptotic cell death, reduced ROS production, and decreased lipid peroxidation induced by MPP+ (Li et al., 2013). In the same vein, a recent report shows that the pharmacological or transcriptional inhibition of SOCE proteins protects against oxidative glutamate toxicity and against MPP+ injury (Maher et al., 2018).

**FIGURE 3:**
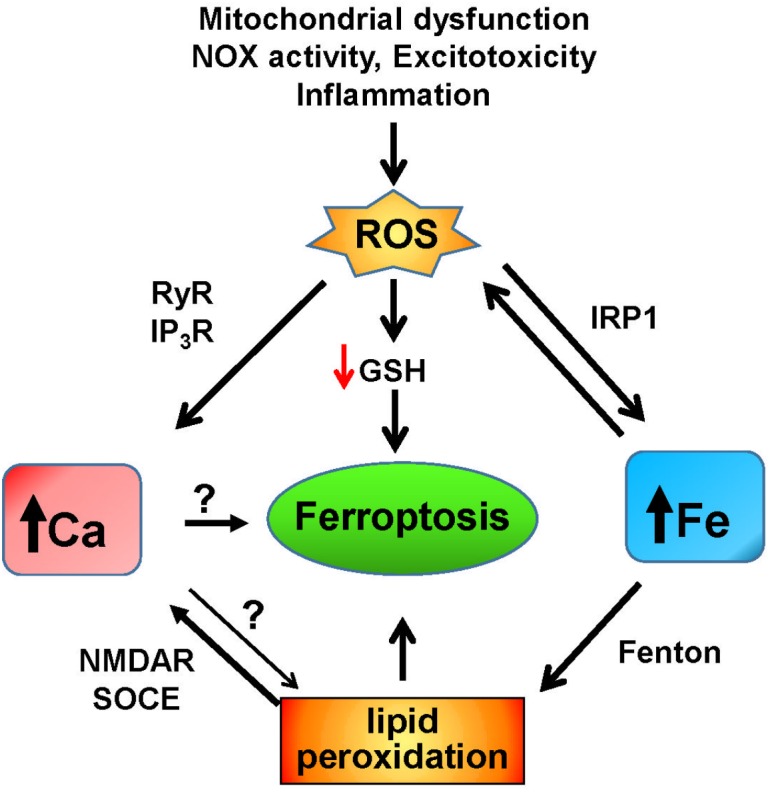
Iron and calcium-mediated cell death by ferroptosis. Increases in ROS due to mitochondrial dysfunction, non-physiological activation of NOX, excitotoxicity, inflammation, or other factors result in the increase of redox-active iron mediated by activation of IRP1. Through the Fenton reaction, iron induces lipid peroxidation that activates NMDAR and the SOCE system, which results in increased cytoplasmic calcium. Increased iron levels and lipid peroxidation mediate ferroptotic cell death, which may also be induced by unregulated calcium levels. Increased ROS levels decrease the intracellular GSH content, leading to ferroptosis, and activate the intracellular calcium channels RyR and IP3R, which adds to the increase in cytoplasmic calcium caused by iron-induced lipid peroxidation.

Overall, these results point to a possible participation of calcium in ferroptosis, a participation driven by raises in cytoplasmic calcium because of oxidative- and lipid peroxidation-derived modifications of the calcium homeostasis machinery (Figure 3). This participation may be specific to cells with weak antioxidant defenses, which could allow for the oxidative activation of calcium channels.

## Protection of Iron Overload by Nrf2; the Calcium Connection

Under oxidative stress conditions, the transcription factor known as nuclear factor (erythroid-derived 2)-like 2 (Nrf2) directs the expression of a large array of cytoprotective genes (Tonelli et al., 2018). In particular, Nrf2 protects cells from the injurious effects of iron overload by regulating the expression of genes involved in iron storage and iron export (Chorley et al., 2012; Kerins and Ooi, 2018). These findings raise the possibility that Nrf2 may counteract the oxidative stress induced in neuronal cells by iron overload. Thus, Nrf2 may restore the intracellular labile iron pool by inducing the expression of the iron exporter FPN1 and ferritin, which oxidizes Fe^2+^ to Fe^3+^ and stores it within its structure making it unavailable for the Fenton reaction (Orino et al., 2001). In addition, intracellular iron levels act as a link between HIF-1α and Nrf2: Nrf2-induced ferritin upregulation activates HIF-1α by making iron unavailable to HIF prolyl-hydroxylase (Siegert et al., 2015), whereas Nrf2 inhibition results in decreased activation of HIF-1α by hypoxia (Kim et al., 2011; Ji et al., 2014).

Recent studies point to a connection between ROS, cellular calcium signals, and Nrf2. The neurotrophin BDNF, besides inducing complex neuronal signaling cascades underlying synaptic plasticity (Bolton et al., 2000), also induces neuronal antioxidant responses through BDNF-induced Nrf2 nuclear translocation (Bouvier et al., 2017; Bruna et al., 2018). In this regard, a recent study showed that BDNF-mediated activation of Nrf2 in astrocytes, which is under circadian control, protects dopaminergic neurons from ferroptosis (Ishii et al., 2018). Of note, in primary hippocampal neurons, ROS and RyR-mediated calcium signals are required for BDNF-induced nuclear translocation of Nrf2 (Bruna et al., 2018). It remains to be established if iron overload in hippocampal neurons engages BDNF and RyR-mediated calcium signals to promote Nrf2-induced expression of protective genes.

## Conclusion

**FIGURE 4:**
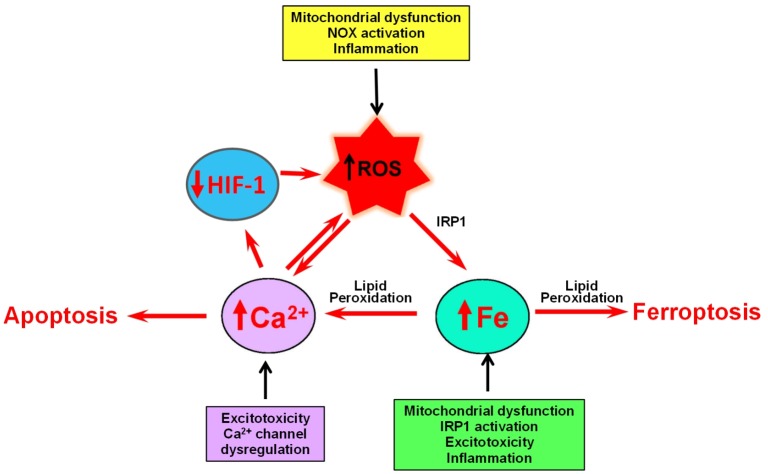
The self-feeding cycle ROS–Fe–Ca. Mitochondrial dysfunction promotes in increased ROS and activation IRP1, which results in increased iron accumulation. Excess iron promotes lipid peroxidation by hydroxyl radicals derived from the Fenton reaction. Lipid peroxidation modifies the activity of a variety of proteins involved in calcium homeostasis that results in massive calcium influx. Increased cytoplasmic calcium levels result in increased mitochondrial calcium and accentuated mitochondrial dysfunction, oxidative stress, and damage. If uncontrolled, this ROS–iron–calcium self-feeding cycle becomes deleterious to mitochondrial and neuronal function, leading eventually to apoptotic or ferroptotic cell death. In yet another aspect of this cycle, high intracellular calcium levels increase the rate of HIF-1 proteasome degradation, which results in increased ROS levels, product of decreased expression of antioxidant proteins.

This article reviews the little acknowledged relationship between iron and calcium in neurodegeneration. The main premise of this article is that increased ROS production generated by iron accumulation profoundly influences calcium homeostasis and signaling (Figure 4).

The influence of increased ROS is clearly shown in the process of oxytosis, in which lipid peroxidation initiated by Fenton-derived hydroxyl radicals results in the modification of a variety of proteins involved in calcium homeostasis that brings to calcium upsurges and cell death. Increased cytoplasmic calcium levels caused by iron dysregulation result in increased mitochondrial calcium and accentuated oxidative stress and damage. If uncontrolled, this calcium/iron self-feeding cycle becomes deleterious to mitochondrial and neuronal function, leading eventually to neuronal death. In yet another aspect of neurodegeneration, high intracellular calcium levels increase the rate of HIF-1 proteasomal degradation while high levels of iron both maintain the activity of HIF-1 hydrolases and, through oxidative stress increase the cellular levels of calcium by activation of ER and PMCA channels. The main conclusion we draw from this review is that dysregulated iron or calcium levels promote deleterious crosstalk between iron and calcium that can result in neuronal dysfunction and death. To our knowledge, this is the first attempt in literature to systemize this important relationship.

## Author Contributions

MN and CH wrote this article.

## Conflict of Interest Statement

The authors declare that the research was conducted in the absence of any commercial or financial relationships that could be construed as a potential conflict of interest.

## References

[B1] Alzheimer’s Association Calcium Hypothesis Workgroup [AACHW] (2017). Calcium hypothesis of Alzheimer’s disease and brain aging: a framework for integrating new evidence into a comprehensive theory of pathogenesis. *Alzheimers Dement.* 13 178.e17–182.e17. 10.1016/j.jalz.2016.12.006 28061328

[B2] An H. Zeng X. Niu T. Li G. Yang J. Zheng L. (2018). Quantifying iron deposition within the substantia nigra of Parkinson’s disease by quantitative susceptibility mapping. *J. Neurol. Sci.* 386 46–52. 10.1016/j.jns.2018.01.008 29406966

[B3] Angelova P. R. Abramov A. Y. (2017). Alpha-synuclein and beta-amyloid - different targets, same players: calcium, free radicals and mitochondria in the mechanism of neurodegeneration. *Biochem. Biophys. Res. Commun.* 483 1110–1115. 10.1016/j.bbrc.2016.07.103 27470584

[B4] Ansari M. A. Scheff S. W. (2011). NADPH-oxidase activation and cognition in Alzheimer disease progression. *Free Radic. Biol. Med.* 51 171–178. 10.1016/j.freeradbiomed.2011.03.025 21457777 PMC3109185

[B5] Area-Gomez E. Schon E. A. (2017). On the pathogenesis of Alzheimer’s Disease: the MAM hypothesis. *FASEB J.* 31 864–867. 10.1096/fj.201601309 28246299 PMC6191063

[B6] Arruda S. F. Siqueira E. M. De Valencia F. F. (2009). Vitamin A deficiency increases hepcidin expression and oxidative stress in rat. *Nutrition* 25 472–478. 10.1016/j.nut.2008.11.030 19217259

[B7] Asiimwe N. Yeo S. G. Kim M. S. Jung J. Jeong N. Y. (2016). Nitric oxide: exploring the contextual link with alzheimer’s disease. *Oxid. Med. Cell. Longev.* 2016:7205747. 10.1155/2016/7205747 28096943 PMC5209623

[B8] Bading H. (2013). Nuclear calcium signalling in the regulation of brain function. *Nat. Rev. Neurosci.* 14 593–608. 10.1038/nrn3531 23942469

[B9] Barrett J. P. Henry R. J. Villapol S. Stoica B. A. Kumar A. Burns M. P. (2017). NOX2 deficiency alters macrophage phenotype through an IL-10/STAT3 dependent mechanism: implications for traumatic brain injury. *J. Neuroinflammation* 14:65. 10.1186/s12974-017-0843-4 28340575 PMC5366128

[B10] Baumgartel K. Mansuy I. M. (2012). Neural functions of calcineurin in synaptic plasticity and memory. *Learn. Mem.* 19 375–384. 10.1101/lm.027201.112 22904368

[B11] Becerril-Ortega J. Bordji K. Freret T. Rush T. Buisson A. (2014). Iron overload accelerates neuronal amyloid-beta production and cognitive impairment in transgenic mice model of Alzheimer’s disease. *Neurobiol. Aging* 35 2288–2301. 10.1016/j.neurobiolaging.2014.04.019 24863668

[B12] Berchner-Pfannschmidt U. Petrat F. Doege K. Trinidad B. Freitag P. Metzen E. (2004). Chelation of cellular calcium modulates hypoxia-inducible gene expression through activation of hypoxia-inducible factor-1alpha. *J. Biol. Chem.* 279 44976–44986. 10.1074/jbc.M313995200 15322093

[B13] Berg D. Youdim M. B. (2006). Role of iron in neurodegenerative disorders. *Top. Magn. Reson. Imaging* 17 5–17. 10.1097/01.rmr.0000245461.90406.ad 17179893

[B14] Berridge M. J. (1998). Neuronal calcium signaling. *Neuron* 21 13–26. 10.1016/S0896-6273(00)80510-3 9697848

[B15] Berridge M. J. Bootman M. D. Roderick H. L. (2003). Calcium signalling: dynamics, homeostasis and remodelling. *Nat. Rev. Mol. Cell Biol.* 4 517–529. 10.1038/nrm1155 12838335

[B16] Berrocal M. Corbacho I. Vazquez-Hernandez M. Avila J. Sepulveda M. R. Mata A. M. (2015). Inhibition of PMCA activity by tau as a function of aging and Alzheimer’s neuropathology. *Biochim. Biophys. Acta* 1852 1465–1476. 10.1016/j.bbadis.2015.04.007 25892185

[B17] Bezprozvanny I. Mattson M. P. (2008). Neuronal calcium mishandling and the pathogenesis of Alzheimer’s disease. *Trends Neurosci.* 31 454–463. 10.1016/j.tins.2008.06.005 18675468 PMC2566585

[B18] Bindokas V. P. Jordan J. Lee C. C. Miller R. J. (1996). Superoxide production in rat hippocampal neurons: selective imaging with hydroethidine. *J. Neurosci.* 16 1324–1336. 10.1523/JNEUROSCI.16-04-01324.1996 8778284 PMC6578569

[B19] Bolton M. M. Pittman A. J. Lo D. C. (2000). Brain-derived neurotrophic factor differentially regulates excitatory and inhibitory synaptic transmission in hippocampal cultures. *J. Neurosci.* 20 3221–3232. 10.1523/JNEUROSCI.20-09-03221.2000 10777787 PMC6773110

[B20] Bors W. Buettner G. R. Michel C. Saran M. (1990). Calcium in lipid peroxidation: does calcium interact with superoxide? *Arch. Biochem. Biophys.* 278 269–272. 10.1016/0003-9861(90)90258-Z 2157359

[B21] Bose A. Beal M. F. (2016). Mitochondrial dysfunction in Parkinson’s disease. *J. Neurochem.* 139(Suppl. 1), 216–231. 10.1111/jnc.13731 27546335

[B22] Bostanci M. O. Bagirici F. (2013). Blocking of L-type calcium channels protects hippocampal and nigral neurons against iron neurotoxicity. The role of L-type calcium channels in iron-induced neurotoxicity. *Int. J. Neurosci.* 123 876–882. 10.3109/00207454.2013.813510 23768064

[B23] Bouvier E. Brouillard F. Molet J. Claverie D. Cabungcal J. H. Cresto N. (2017). Nrf2-dependent persistent oxidative stress results in stress-induced vulnerability to depression. *Mol. Psychiatry* 22 1701–1713. 10.1038/mp.2016.211 27646262

[B24] Braughler J. M. Duncan L. A. Chase R. L. (1985). Interaction of lipid peroxidation and calcium in the pathogenesis of neuronal injury. *Cent. Nerv. Syst. Trauma* 2 269–283. 10.1089/cns.1985.2.269 2424624

[B25] Brennan A. M. Suh S. W. Won S. J. Narasimhan P. Kauppinen T. M. Lee H. (2009). NADPH oxidase is the primary source of superoxide induced by NMDA receptor activation. *Nat. Neurosci.* 12 857–863. 10.1038/nn.2334 19503084 PMC2746760

[B26] Brini M. Cali T. Ottolini D. Carafoli E. (2014). Neuronal calcium signaling: function and dysfunction. *Cell. Mol. Life Sci.* 71 2787–2814. 10.1007/s00018-013-1550-7 24442513 PMC11113927

[B27] Brini M. Leanza L. Szabo I. (2017). Lipid-mediated modulation of intracellular ion channels and redox state: physiopathological implications. *Antioxid. Redox Signal.* 10.1089/ars.2017.7215 [Epub ahead of print]. 28679281

[B28] Bruick R. K. McKnight S. L. (2001). A conserved family of prolyl-4-hydroxylases that modify HIF. *Science* 294 1337–1340. 10.1126/science.1066373 11598268

[B29] Bruna B. Lobos P. Herrera-Molina R. Hidalgo C. Paula-Lima A. Adasme T. (2018). The signaling pathways underlying BDNF-induced Nrf2 hippocampal nuclear translocation involve ROS, RyR-Mediated Ca(2+) signals, ERK and PI3K. *Biochem. Biophys. Res. Commun.* 505 201–207. 10.1016/j.bbrc.2018.09.080 30243728

[B30] Bulk M. Van Der Weerd L. Breimer W. Lebedev N. Webb A. Goeman J. J. (2018). Quantitative comparison of different iron forms in the temporal cortex of Alzheimer patients and control subjects. *Sci. Rep.* 8:6898. 10.1038/s41598-018-25021-7 29720594 PMC5932027

[B31] Burbulla L. F. Song P. Mazzulli J. R. Zampese E. Wong Y. C. Jeon S. (2017). Dopamine oxidation mediates mitochondrial and lysosomal dysfunction in Parkinson’s disease. *Science* 357 1255–1261. 10.1126/science.aam9080 28882997 PMC6021018

[B32] Cabantchik Z. I. (2014). Labile iron in cells and body fluids: physiology, pathology, and pharmacology. *Front. Pharmacol.* 5:45 10.3389/fphar.2014.00045 PMC3952030 24659969

[B33] Caltagirone A. Weiss G. Pantopoulos K. (2001). Modulation of cellular iron metabolism by hydrogen peroxide. Effects of H2O2 on the expression and function of iron-responsive element-containing mRNAs in B6 fibroblasts. *J. Biol. Chem.* 276 19738–19745. 10.1074/jbc.M100245200 11264285

[B34] Cannon J. R. Tapias V. Na H. M. Honick A. S. Drolet R. E. Greenamyre J. T. (2009). A highly reproducible rotenone model of Parkinson’s disease. *Neurobiol. Dis.* 34 279–290. 10.1016/j.nbd.2009.01.016 19385059 PMC2757935

[B35] Carocci A. Catalano A. Sinicropi M. S. Genchi G. (2018). Oxidative stress and neurodegeneration: the involvement of iron. *Biometals* 31 715–735. 10.1007/s10534-018-0126-2 30014355

[B36] Chan C. S. Gertler T. S. Surmeier D. J. (2009). Calcium homeostasis, selective vulnerability and Parkinson’s disease. *Trends Neurosci.* 32 249–256. 10.1016/j.tins.2009.01.006 19307031 PMC4831702

[B37] Cheah J. H. Kim S. F. Hester L. D. Clancy K. W. Patterson S. E. III Papadopoulos V. (2006). NMDA receptor-nitric oxide transmission mediates neuronal iron homeostasis via the GTPase Dexras1. *Neuron* 51 431–440. 10.1016/j.neuron.2006.07.011 16908409 PMC3150500

[B38] Chinopoulos C. Adam-Vizi V. (2006). Calcium, mitochondria and oxidative stress in neuronal pathology. Novel aspects of an enduring theme. *FEBS J.* 273 433–450. 10.1111/j.1742-4658.2005.05103.x 16420469

[B39] Chorley B. N. Campbell M. R. Wang X. Karaca M. Sambandan D. Bangura F. (2012). Identification of novel NRF2-regulated genes by ChIP-Seq: influence on retinoid X receptor alpha. *Nucleic Acids Res.* 40 7416–7429. 10.1093/nar/gks409 22581777 PMC3424561

[B40] Clapham D. E. (2007). Calcium signaling. *Cell* 131 1047–1058. 10.1016/j.cell.2007.11.028 18083096

[B41] Cobley J. N. Fiorello M. L. Bailey D. M. (2018). 13 reasons why the brain is susceptible to oxidative stress. *Redox Biol.* 15 490–503. 10.1016/j.redox.2018.01.008 29413961 PMC5881419

[B42] Creighton-Gutteridge M. Tyrrell R. M. (2002). A novel iron chelator that does not induce HIF-1 activity. *Free Radic. Biol. Med.* 33 356–363. 10.1016/S0891-5849(02)00884-5 12126757

[B43] Crichton R. R. Dexter D. T. Ward R. J. (2011). Brain iron metabolism and its perturbation in neurological diseases. *J. Neural Transm.* 118 301–314. 10.1007/s00702-010-0470-z 20809066

[B44] Cristovao J. S. Santos R. Gomes C. M. (2016). Metals and neuronal metal binding proteins implicated in Alzheimer’s Disease. *Oxid. Med. Cell. Longev.* 2016:9812178. 10.1155/2016/9812178 26881049 PMC4736980

[B45] Damasceno F. C. Condeles A. L. Lopes A. K. B. Facci R. R. Linares E. Truzzi D. R. (2018). The labile iron pool attenuates peroxynitrite-dependent damage and can no longer be considered solely a pro-oxidative cellular iron source. *J. Biol. Chem.* 293 8530–8542. 10.1074/jbc.RA117.000883 29661935 PMC5986223

[B46] Davies M. J. (2005). The oxidative environment and protein damage. *Biochim. Biophys. Acta* 1703 93–109. 10.1016/j.bbapap.2004.08.007 15680218

[B47] Demuro A. Parker I. Stutzmann G. E. (2010). Calcium signaling and amyloid toxicity in Alzheimer disease. *J. Biol. Chem.* 285 12463–12468. 10.1074/jbc.R109.080895 20212036 PMC2857063

[B48] Dev S. Kumari S. Singh N. Kumar Bal S. Seth P. Mukhopadhyay C. K. (2015). Role of extracellular hydrogen peroxide in regulation of iron homeostasis genes in neuronal cells: implication in iron accumulation. *Free Radic. Biol. Med.* 86 78–89. 10.1016/j.freeradbiomed.2015.05.025 26006106

[B49] Di Lisa F. Bernardi P. (2009). A CaPful of mechanisms regulating the mitochondrial permeability transition. *J. Mol. Cell Cardiol.* 46 775–780. 10.1016/j.yjmcc.2009.03.006 19303419

[B50] DiSabato D. J. Quan N. Godbout J. P. (2016). Neuroinflammation: the devil is in the details. *J. Neurochem.* 139(Suppl. 2), 136–153. 10.1111/jnc.13607 26990767 PMC5025335

[B51] Dixon S. J. Lemberg K. M. Lamprecht M. R. Skouta R. Zaitsev E. M. Gleason C. E. (2012). Ferroptosis: an iron-dependent form of nonapoptotic cell death. *Cell* 149 1060–1072. 10.1016/j.cell.2012.03.042 22632970 PMC3367386

[B52] Dixon S. J. Stockwell B. R. (2014). The role of iron and reactive oxygen species in cell death. *Nat. Chem. Biol.* 10 9–17. 10.1038/nchembio.1416 24346035

[B53] Drapier J. C. Hirling H. Wietzerbin J. Kaldy P. Kuhn L. C. (1993). Biosynthesis of nitric oxide activates iron regulatory factor in macrophages. *EMBO J.* 12 3643–3649. 10.1002/j.1460-2075.1993.tb06038.x 7504626 PMC413640

[B54] Duncan A. J. Heales S. J. (2005). Nitric oxide and neurological disorders. *Mol. Aspects Med.* 26 67–96. 10.1016/j.mam.2004.09.004 15722115

[B55] Flagg S. C. Martin C. B. Taabazuing C. Y. Holmes B. E. Knapp M. J. (2012). Screening chelating inhibitors of HIF-prolyl hydroxylase domain 2 (PHD2) and factor inhibiting HIF (FIH). *J. Inorg. Biochem.* 113 25–30. 10.1016/j.jinorgbio.2012.03.002 22687491 PMC3525482

[B56] Franco-Iborra S. Vila M. Perier C. (2018). Mitochondrial quality control in neurodegenerative diseases: focus on parkinson’s disease and huntington’s disease. *Front. Neurosci.* 12:342 10.3389/fnins.2018.00342 PMC5974257 29875626

[B57] Fricker M. Tolkovsky A. M. Borutaite V. Coleman M. Brown G. C. (2018). Neuronal cell death. *Physiol. Rev.* 98 813–880. 10.1152/physrev.00011.2017 29488822 PMC5966715

[B58] Fukui K. (2016). Reactive oxygen species induce neurite degeneration before induction of cell death. *J. Clin. Biochem. Nutr.* 59 155–159. 10.3164/jcbn.16-34 27895381 PMC5110939

[B59] Gaasch J. A. Geldenhuys W. J. Lockman P. R. Allen D. D. Van Der Schyf C. J. (2007). Voltage-gated calcium channels provide an alternate route for iron uptake in neuronal cell cultures. *Neurochem. Res.* 32 1686–1693. 10.1007/s11064-007-9313-1 17404834

[B60] Gao H. M. Zhou H. Hong J. S. (2012). NADPH oxidases: novel therapeutic targets for neurodegenerative diseases. *Trends Pharmacol. Sci.* 33 295–303. 10.1016/j.tips.2012.03.008 22503440 PMC3477578

[B61] Garrick M. D. Singleton S. T. Vargas F. Kuo H. C. Zhao L. Knopfel M. (2006). DMT1: which metals does it transport? *Biol. Res.* 39 79–85. 10.4067/S0716-97602006000100009 16629167

[B62] Ghosh D. K. Salerno J. C. (2003). Nitric oxide synthases: domain structure and alignment in enzyme function and control. *Front. Biosci.* 8 D193–D209. 10.2741/959 12456347

[B63] Gibson G. E. Thakkar A. (2017). Interactions of mitochondria/metabolism and calcium regulation in alzheimer’s disease: a calcinist point of view. *Neurochem. Res.* 42 1636–1648. 10.1007/s11064-017-2182-3 28181072 PMC5451308

[B64] Giorgi C. Baldassari F. Bononi A. Bonora M. De Marchi E. Marchi S. (2012). Mitochondrial Ca(2+) and apoptosis. *Cell Calcium* 52 36–43. 10.1016/j.ceca.2012.02.008 22480931 PMC3396846

[B65] Giorgi C. Marchi S. Pinton P. (2018). The machineries, regulation and cellular functions of mitochondrial calcium. *Nat. Rev. Mol. Cell Biol.* 19 713–730. 10.1038/s41580-018-0052-8 30143745

[B66] Girouard H. Wang G. Gallo E. F. Anrather J. Zhou P. Pickel V. M. (2009). NMDA receptor activation increases free radical production through nitric oxide and NOX2. *J. Neurosci.* 29 2545–2552. 10.1523/JNEUROSCI.0133-09.2009 19244529 PMC2669930

[B67] Gonsette R. E. (2008). Neurodegeneration in multiple sclerosis: the role of oxidative stress and excitotoxicity. *J. Neurol. Sci.* 274 48–53. 10.1016/j.jns.2008.06.029 18684473

[B68] Gordeeva A. V. Zvyagilskaya R. A. Labas Y. A. (2003). Cross-talk between reactive oxygen species and calcium in living cells. *Biochemistry* 68 1077–1080. 14616077 10.1023/a:1026398310003

[B69] Gorlach A. Bertram K. Hudecova S. Krizanova O. (2015). Calcium and ROS: a mutual interplay. *Redox Biol.* 6 260–271. 10.1016/j.redox.2015.08.010 26296072 PMC4556774

[B70] Green S. P. Cairns B. Rae J. Errett-Baroncini C. Hongo J. A. Erickson R. W. (2001). Induction of gp91-phox, a component of the phagocyte NADPH oxidase, in microglial cells during central nervous system inflammation. *J. Cereb. Blood Flow Metab.* 21 374–384. 10.1097/00004647-200104000-00006 11323523

[B71] Grosskreutz J. Van Den Bosch L. Keller B. U. (2010). Calcium dysregulation in amyotrophic lateral sclerosis. *Cell Calcium* 47 165–174. 10.1016/j.ceca.2009.12.002 20116097

[B72] Guo C. Hao L. J. Yang Z. H. Chai R. Zhang S. Gu Y. (2016). Deferoxamine-mediated up-regulation of HIF-1alpha prevents dopaminergic neuronal death via the activation of MAPK family proteins in MPTP-treated mice. *Exp. Neurol.* 280 13–23. 10.1016/j.expneurol.2016.03.016 26996132

[B73] Guo C. Zhang Y. X. Wang T. Zhong M. L. Yang Z. H. Hao L. J. (2015). Intranasal deferoxamine attenuates synapse loss via up-regulating the P38/HIF-1alpha pathway on the brain of APP/PS1 transgenic mice. *Front. Aging Neurosci.* 7:104. 10.3389/fnagi.2015.00104 26082716 PMC4451419

[B74] Guo J. D. Zhao X. Li Y. Li G. R. Liu X. L. (2018). Damage to dopaminergic neurons by oxidative stress in Parkinson’s disease (Review). *Int. J. Mol. Med.* 41 1817–1825. 10.3892/ijmm.2018.3406 29393357

[B75] Guzman J. N. Sanchez-Padilla J. Chan C. S. Surmeier D. J. (2009). Robust pacemaking in substantia nigra dopaminergic neurons. *J. Neurosci.* 29 11011–11019. 10.1523/JNEUROSCI.2519-09.2009 19726659 PMC2784968

[B76] Hajieva P. Baeken M. W. Moosmann B. (2018). The role of plasma membrane calcium atpases (PMCAS) in neurodegenerative disorders. *Neurosci. Lett.* 663 29–38. 10.1016/j.neulet.2017.09.033 29452613

[B77] Halliwell B. (2006). Oxidative stress and neurodegeneration: where are we now? *J. Neurochem.* 97 1634–1658. 10.1111/j.1471-4159.2006.03907.x 16805774

[B78] Hambright W. S. Fonseca R. S. Chen L. Na R. Ran Q. (2017). Ablation of ferroptosis regulator glutathione peroxidase 4 in forebrain neurons promotes cognitive impairment and neurodegeneration. *Redox Biol.* 12 8–17. 10.1016/j.redox.2017.01.021 28212525 PMC5312549

[B79] Hanke M. L. Kielian T. (2011). Toll-like receptors in health and disease in the brain: mechanisms and therapeutic potential. *Clin. Sci.* 121 367–387. 10.1042/CS20110164 21745188 PMC4231819

[B80] Hanson E. S. Leibold E. A. (1999). Regulation of the iron regulatory proteins by reactive nitrogen and oxygen species. *Gene Expr.* 7 367–376. 10440237 PMC6174660

[B81] Hansson Petersen C. A. Alikhani N. Behbahani H. Wiehager B. Pavlov P. F. Alafuzoff I. (2008). The amyloid beta-peptide is imported into mitochondria via the TOM import machinery and localized to mitochondrial cristae. *Proc. Natl. Acad. Sci. U.S.A.* 105 13145–13150. 10.1073/pnas.0806192105 18757748 PMC2527349

[B82] Hepp S. Gerich F. J. Muller M. (2005). Sulfhydryl oxidation reduces hippocampal susceptibility to hypoxia-induced spreading depression by activating BK channels. *J. Neurophysiol.* 94 1091–1103. 10.1152/jn.00291.2005 15872065

[B83] Hewitson K. S. Mcneill L. A. Elkins J. M. Schofield C. J. (2003). The role of iron and 2-oxoglutarate oxygenases in signalling. *Biochem. Soc. Trans.* 31 510–515. 10.1042/bst0310510 12773146

[B84] Hidalgo C. Arias-Cavieres A. (2016). Calcium, reactive oxygen species, and synaptic plasticity. *Physiology* 31 201–215. 10.1152/physiol.00038.2015 27053734

[B85] Hidalgo C. Carrasco M. A. Muñoz P. Núñez M. T. (2007). A role for reactive oxygen/nitrogen species and iron on neuronal synaptic plasticity. *Antioxid. Redox. Signal.* 9 245–255. 10.1089/ars.2007.9.245 17115937

[B86] Hidalgo C. Donoso P. (2008). Crosstalk between calcium and redox signaling: from molecular mechanisms to health implications. *Antioxid. Redox Signal.* 10 1275–1312. 10.1089/ars.2007.1886 18377233

[B87] Hidalgo C. Núñez M. T. (2007). Calcium, iron and neuronal function. *IUBMB Life* 59 280–285. 10.1080/15216540701222906 17505966

[B88] Huang M. L. Lane D. J. Richardson D. R. (2011). Mitochondrial mayhem: the mitochondrion as a modulator of iron metabolism and its role in disease. *Antioxid. Redox Signal.* 15 3003–3019. 10.1089/ars.2011.3921 21545274

[B89] Huang X. Atwood C. S. Hartshorn M. A. Multhaup G. Goldstein L. E. Scarpa R. C. (1999). The A beta peptide of Alzheimer’s disease directly produces hydrogen peroxide through metal ion reduction. *Biochemistry* 38 7609–7616. 10.1021/bi990438f 10386999

[B90] Ishii T. Warabi E. Mann G. E. (2018). Circadian control of BDNF-mediated Nrf2 activation in astrocytes protects dopaminergic neurons from ferroptosis. *Free Radic. Biol. Med.* 10.1016/j.freeradbiomed.2018.09.002 [Epub ahead of print]. 30189266

[B91] Jaiswal M. K. (2013). Calcium, mitochondria, and the pathogenesis of ALS: the good, the bad, and the ugly. *Front. Cell. Neurosci.* 7:199. 10.3389/fncel.2013.00199 24198760 PMC3813898

[B92] Jellinger K. A. (1999). The role of iron in neurodegeneration: prospects for pharmacotherapy of Parkinson’s disease. *Drugs Aging* 14 115–140. 10.2165/00002512-199914020-00004 10084365

[B93] Jeney V. Balla J. Yachie A. Varga Z. Vercellotti G. M. Eaton J. W. (2002). Pro-oxidant and cytotoxic effects of circulating heme. *Blood* 100 879–887. 10.1182/blood.V100.3.879 12130498

[B94] Jeong S. Y. Rathore K. I. Schulz K. Ponka P. Arosio P. David S. (2009). Dysregulation of iron homeostasis in the CNS contributes to disease progression in a mouse model of amyotrophic lateral sclerosis. *J. Neurosci.* 29 610–619. 10.1523/JNEUROSCI.5443-08.2009 19158288 PMC6665152

[B95] Ji X. Wang H. Zhu J. Zhu L. Pan H. Li W. (2014). Knockdown of Nrf2 suppresses glioblastoma angiogenesis by inhibiting hypoxia-induced activation of HIF-1alpha. *Int. J. Cancer* 135 574–584. 10.1002/ijc.28699 24374745

[B96] Jiang L. Bechtel M. D. Galeva N. A. Williams T. D. Michaelis E. K. Michaelis M. L. (2012). Decreases in plasma membrane Ca(2)(+)-ATPase in brain synaptic membrane rafts from aged rats. *J. Neurochem.* 123 689–699. 10.1111/j.1471-4159.2012.07918.x 22889001 PMC3493797

[B97] Joseph S. K. Young M. P. Alzayady K. Yule D. I. Ali M. Booth D. M. (2018). Redox regulation of type-I inositol trisphosphate receptors in intact mammalian cells. *J. Biol. Chem.* 293 17464–17476. 10.1074/jbc.RA118.005624 30228182 PMC6231128

[B98] Kahlert S. Zundorf G. Reiser G. (2005). Glutamate-mediated influx of extracellular Ca2+ is coupled with reactive oxygen species generation in cultured hippocampal neurons but not in astrocytes. *J. Neurosci. Res.* 79 262–271. 10.1002/jnr.20322 15578732

[B99] Kasarskis E. J. Tandon L. Lovell M. A. Ehmann W. D. (1995). Aluminum, calcium, and iron in the spinal cord of patients with sporadic amyotrophic lateral sclerosis using laser microprobe mass spectroscopy: a preliminary study. *J. Neurol. Sci.* 130 203–208. 10.1016/0022-510X(95)00037-3 8586987

[B100] Kastanenka K. V. Bussiere T. Shakerdge N. Qian F. Weinreb P. H. Rhodes K. (2016). Immunotherapy with aducanumab restores calcium homeostasis in Tg2576 Mice. *J. Neurosci.* 36 12549–12558. 10.1523/JNEUROSCI.2080-16.2016 27810931 PMC5157102

[B101] Kerins M. J. Ooi A. (2018). The roles of NRF2 in modulating cellular iron homeostasis. *Antioxid. Redox Signal.* 29 1756–1773. 10.1089/ars.2017.7176 28793787 PMC6208163

[B102] Kietzmann T. Knabe W. Schmidt-Kastner R. (2001). Hypoxia and hypoxia-inducible factor modulated gene expression in brain: involvement in neuroprotection and cell death. *Eur. Arch. Psychiatry Clin. Neurosci.* 251 170–178. 10.1007/s004060170037 11697581

[B103] Kim T. H. Hur E. G. Kang S. J. Kim J. A. Thapa D. Lee Y. M. (2011). NRF2 blockade suppresses colon tumor angiogenesis by inhibiting hypoxia-induced activation of HIF-1alpha. *Cancer Res.* 71 2260–2275. 10.1158/0008-5472.CAN-10-3007 21278237

[B104] Kishida K. T. Klann E. (2007). Sources and targets of reactive oxygen species in synaptic plasticity and memory. *Antioxid. Redox Signal.* 9 233–244. 10.1089/ars.2007.9.233 17115936 PMC3544198

[B105] Kupershmidt L. Weinreb O. Amit T. Mandel S. Bar-Am O. Youdim M. B. (2011). Novel molecular targets of the neuroprotective/neurorescue multimodal iron chelating drug M30 in the mouse brain. *Neuroscience* 189 345–358. 10.1016/j.neuroscience.2011.03.040 21570450

[B106] Kupershmidt L. Weinreb O. Amit T. Mandel S. Carri M. T. Youdim M. B. (2009). Neuroprotective and neuritogenic activities of novel multimodal iron-chelating drugs in motor-neuron-like NSC-34 cells and transgenic mouse model of amyotrophic lateral sclerosis. *FASEB J.* 23 3766–3779. 10.1096/fj.09-130047 19638399

[B107] Kushnir A. Wajsberg B. Marks A. R. (2018). Ryanodine receptor dysfunction in human disorders. *Biochim. Biophys. Acta Mol. Cell. Res.* 10.1016/j.bbamcr.2018.07.011 [Epub ahead of print]. 30040966

[B108] Lane D. J. R. Ayton S. Bush A. I. (2018). Iron and Alzheimer’s disease: an update on emerging mechanisms. *J. Alzheimers Dis.* 64 S379–S395. 10.3233/JAD-179944 29865061

[B109] Lee D. G. Park J. Lee H. S. Lee S. R. Lee D. S. (2016). Iron overload-induced calcium signals modulate mitochondrial fragmentation in HT-22 hippocampal neuron cells. *Toxicology* 365 17–24. 10.1016/j.tox.2016.07.022 27481217

[B110] Lee D. W. Andersen J. K. (2006). Role of HIF-1 in iron regulation: potential therapeutic strategy for neurodegenerative disorders. *Curr. Mol. Med.* 6 883–893. 10.2174/156652406779010849 17168739

[B111] Lee J. C. Tae H. J. Kim I. H. Cho J. H. Lee T. K. Park J. H. (2017). Roles of HIF-1alpha, VEGF, and NF-kappaB in ischemic preconditioning-mediated neuroprotection of hippocampal CA1 pyramidal neurons against a subsequent transient cerebral ischemia. *Mol. Neurobiol.* 54 6984–6998. 10.1007/s12035-016-0219-2 27785755

[B112] Lee J. H. Han Y. H. Kang B. M. Mun C. W. Lee S. J. Baik S. K. (2013). Quantitative assessment of subcortical atrophy and iron content in progressive supranuclear palsy and parkinsonian variant of multiple system atrophy. *J. Neurol.* 260 2094–2101. 10.1007/s00415-013-6951-x 23670309

[B113] Lewerenz J. Ates G. Methner A. Conrad M. Maher P. (2018). Oxytosis/ferroptosis-(Re-) emerging roles for oxidative stress-dependent non-apoptotic cell death in diseases of the central nervous system. *Front. Neurosci.* 12:214. 10.3389/fnins.2018.00214 29731704 PMC5920049

[B114] Li X. Chen W. Zhang L. Liu W. B. Fei Z. (2013). Inhibition of store-operated calcium entry attenuates MPP(+)-induced oxidative stress via preservation of mitochondrial function in PC12 cells: involvement of Homer1a. *PLoS One* 8:e83638. 10.1371/journal.pone.0083638 24358303 PMC3866123

[B115] Liao Y. Dong Y. Cheng J. (2017). The function of the mitochondrial calcium uniporter in neurodegenerative disorders. *Int. J. Mol. Sci.* 18:E248. 10.3390/ijms18020248 28208618 PMC5343785

[B116] Lim H. Kim D. Lee S. J. (2013). Toll-like receptor 2 mediates peripheral nerve injury-induced NADPH oxidase 2 expression in spinal cord microglia. *J. Biol. Chem.* 288 7572–7579. 10.1074/jbc.M112.414904 23386616 PMC3597798

[B117] Lockman J. A. Geldenhuys W. J. Bohn K. A. Desilva S. F. Allen D. D. Van Der Schyf C. J. (2012). Differential effect of nimodipine in attenuating iron-induced toxicity in brain- and blood-brain barrier-associated cell types. *Neurochem. Res.* 37 134–142. 10.1007/s11064-011-0591-2 21935732

[B118] Lourenco C. F. Ledo A. Barbosa R. M. Laranjinha J. (2017). Neurovascular-neuroenergetic coupling axis in the brain: master regulation by nitric oxide and consequences in aging and neurodegeneration. *Free Radic. Biol. Med.* 108 668–682. 10.1016/j.freeradbiomed.2017.04.026 28435052

[B119] Lu C. Chan S. L. Fu W. Mattson M. P. (2002). The lipid peroxidation product 4-hydroxynonenal facilitates opening of voltage-dependent Ca2+ channels in neurons by increasing protein tyrosine phosphorylation. *J. Biol. Chem.* 277 24368–24375. 10.1074/jbc.M201924200 12006588

[B120] Ludtmann M. H. R. Abramov A. Y. (2018). Mitochondrial calcium imbalance in Parkinson’s disease. *Neurosci. Lett.* 663 86–90. 10.1016/j.neulet.2017.08.044 28838811

[B121] Lynch M. A. (2004). Long-term potentiation and memory. *Physiol. Rev.* 84 87–136. 10.1152/physrev.00014.2003 14715912

[B122] Maher P. Van Leyen K. Dey P. N. Honrath B. Dolga A. Methner A. (2018). The role of Ca(2+) in cell death caused by oxidative glutamate toxicity and ferroptosis. *Cell Calcium* 70 47–55. 10.1016/j.ceca.2017.05.007 28545724 PMC5682235

[B123] Marchi S. Patergnani S. Missiroli S. Morciano G. Rimessi A. Wieckowski M. R. (2018). Mitochondrial and endoplasmic reticulum calcium homeostasis and cell death. *Cell Calcium* 69 62–72. 10.1016/j.ceca.2017.05.003 28515000

[B124] Marro S. Chiabrando D. Messana E. Stolte J. Turco E. Tolosano E. (2010). Heme controls ferroportin1 (FPN1) transcription involving Bach1, Nrf2 and a MARE/ARE sequence motif at position -7007 of the FPN1 promoter. *Haematologica* 95 1261–1268. 10.3324/haematol.2009.020123 20179090 PMC2913073

[B125] Martins E. A. Robalinho R. L. Meneghini R. (1995). Oxidative stress induces activation of a cytosolic protein responsible for control of iron uptake. *Arch. Biochem. Biophys.* 316 128–134. 10.1006/abbi.1995.1019 7840606

[B126] Mata A. M. Sepulveda M. R. (2010). Plasma membrane Ca-ATPases in the nervous system during development and ageing. *World J. Biol. Chem.* 1 229–234. 10.4331/wjbc.v1.i7.229 21537478 PMC3083968

[B127] Mattson M. P. (2010). ER calcium and Alzheimer’s disease: in a state of flux. *Sci. Signal.* 3:e10 10.1126/scisignal.3114pe10 PMC3091478 20332425

[B128] McCarthy R. C. Sosa J. C. Gardeck A. M. Baez A. S. Lee C. H. Wessling-Resnick M. (2018). Inflammation-induced iron transport and metabolism by brain microglia. *J. Biol. Chem.* 293 7853–7863. 10.1074/jbc.RA118.001949 29610275 PMC5961037

[B129] Mechlovich D. Amit T. Bar-Am O. Mandel S. Youdim M. B. Weinreb O. (2014). The novel multi-target iron chelator, M30 modulates HIF-1alpha-related glycolytic genes and insulin signaling pathway in the frontal cortex of APP/PS1 Alzheimer’s disease mice. *Curr. Alzheimer Res.* 11 119–127. 10.2174/1567205010666131212112529 24359498

[B130] Mena N. P. Urrutia P. J. Lourido F. Carrasco C. M. Núñez M. T. (2015). Mitochondrial iron homeostasis and its dysfunctions in neurodegenerative disorders. *Mitochondrion* 21 92–105. 10.1016/j.mito.2015.02.001 25667951

[B131] Merelli A. Rodriguez J. C. G. Folch J. Regueiro M. R. Camins A. Lazarowski A. (2018). Understanding the role of hypoxia inducible factor during neurodegeneration for new therapeutics opportunities. *Curr. Neuropharmacol.* 16 1484–1498. 10.2174/1570159X16666180110130253 29318974 PMC6295932

[B132] Miyamoto M. Murphy T. H. Schnaar R. L. Coyle J. T. (1989). Antioxidants protect against glutamate-induced cytotoxicity in a neuronal cell line. *J. Pharmacol. Exp. Ther.* 250 1132–1140. 2778712

[B133] Moldogazieva N. T. Lutsenko S. V. Terentiev A. A. (2018). Reactive oxygen and nitrogen species-induced protein modifications: implication in carcinogenesis and anticancer therapy. *Cancer Res.* 78 6040–6047. 10.1158/0008-5472.CAN-18-0980 30327380

[B134] Mole D. R. Blancher C. Copley R. R. Pollard P. J. Gleadle J. M. Ragoussis J. (2009). Genome-wide association of hypoxia-inducible factor (HIF)-1alpha and HIF-2alpha DNA binding with expression profiling of hypoxia-inducible transcripts. *J. Biol. Chem.* 284 16767–16775. 10.1074/jbc.M901790200 19386601 PMC2719312

[B135] Moncada S. Bolaños J. P. (2006). Nitric oxide, cell bioenergetics and neurodegeneration. *J. Neurochem.* 97 1676–1689. 10.1111/j.1471-4159.2006.03988.x 16805776

[B136] Mueller S. Pantopoulos K. Hubner C. A. Stremmel W. Hentze M. W. (2001). IRP1 activation by extracellular oxidative stress in the perfused rat liver. *J. Biol. Chem.* 276 23192–23196. 10.1074/jbc.M100654200 11297549

[B137] Muhling T. Duda J. Weishaupt J. H. Ludolph A. C. Liss B. (2014). Elevated mRNA-levels of distinct mitochondrial and plasma membrane Ca(2+) transporters in individual hypoglossal motor neurons of endstage SOD1 transgenic mice. *Front. Cell. Neurosci.* 8:353. 10.3389/fncel.2014.00353 25452714 PMC4231948

[B138] Muñoz P. (2012). Iron-mediated redox modulation in neural plasticity. *Commun. Integr. Biol.* 5 166–168. 10.4161/cib.18710 22808323 PMC3376054

[B139] Muñoz P. Humeres A. Elgueta C. Kirkwood A. Hidalgo C. Núñez M. T. (2011). Iron mediates N-methyl-D-aspartate receptor-dependent stimulation of calcium-induced pathways and hippocampal synaptic plasticity. *J. Biol. Chem.* 286 13382–13392. 10.1074/jbc.M110.213785 21296883 PMC3075684

[B140] Muñoz Y. Carrasco C. M. Campos J. D. Aguirre P. Núñez M. T. (2016). Parkinson’s Disease: the mitochondria-iron link. *Parkinsons Dis.* 2016:7049108. 10.1155/2016/7049108 27293957 PMC4886095

[B141] Muravchick S. Levy R. J. (2006). Clinical implications of mitochondrial dysfunction. *Anesthesiology* 105 819–837. 10.1097/00000542-200610000-00029 17006082

[B142] Murphy T. H. Malouf A. T. Sastre A. Schnaar R. L. Coyle J. T. (1988). Calcium-dependent glutamate cytotoxicity in a neuronal cell line. *Brain Res.* 444 325–332. 10.1016/0006-8993(88)90941-9 2896063

[B143] Murphy T. H. Miyamoto M. Sastre A. Schnaar R. L. Coyle J. T. (1989). Glutamate toxicity in a neuronal cell line involves inhibition of cystine transport leading to oxidative stress. *Neuron* 2 1547–1558. 10.1016/0896-6273(89)90043-3 2576375

[B144] Murray-Kolb L. E. (2013). Iron and brain functions. *Curr. Opin. Clin. Nutr. Metab. Care* 16 703–707. 10.1097/MCO.0b013e3283653ef8 24100670

[B145] Mustaly-Kalimi S. Littlefield A. M. Stutzmann G. E. (2018). Calcium signaling deficits in glia and autophagic pathways contributing to neurodegenerative disease. *Antioxid. Redox Signal.* 29 1158–1175. 10.1089/ars.2017.7266 29634342

[B146] Myllyharju J. (2013). Prolyl 4-hydroxylases, master regulators of the hypoxia response. *Acta Physiol.* 208 148–165. 10.1111/apha.12096 23489300

[B147] Nandal A. Ruiz J. C. Subramanian P. Ghimire-Rijal S. Sinnamon R. A. Stemmler T. L. (2011). Activation of the HIF prolyl hydroxylase by the iron chaperones PCBP1 and PCBP2. *Cell Metab.* 14 647–657. 10.1016/j.cmet.2011.08.015 22055506 PMC3361910

[B148] Nemes R. Koltai E. Taylor A. W. Suzuki K. Gyori F. Radak Z. (2018). Reactive oxygen and nitrogen species regulate key metabolic, anabolic, and catabolic pathways in skeletal muscle. *Antioxidants* 7:E85. 10.3390/antiox7070085 29976853 PMC6071245

[B149] Núñez M. T. (2010). Regulatory mechanisms of intestinal iron absorption-uncovering of a fast-response mechanism based on DMT1 and ferroportin endocytosis. *Biofactors* 36 88–97. 10.1002/biof.84 20232409

[B150] Núñez M. T. Chana-Cuevas P. (2018). New perspectives in iron chelation therapy for the treatment of neurodegenerative diseases. *Pharmaceuticals* 11:E109. 10.3390/ph11040109 30347635 PMC6316457

[B151] Núñez M. T. Gallardo V. Muñoz P. Tapia V. Esparza A. Salazar J. (2004). Progressive iron accumulation induces a biphasic change in the glutathione content of neuroblastoma cells. *Free Radic. Biol. Med.* 37 953–960. 10.1016/j.freeradbiomed.2004.06.005 15336311

[B152] Núñez M. T. Urrutia P. Mena N. Aguirre P. Tapia V. Salazar J. (2012). Iron toxicity in neurodegeneration. *Biometals* 25 761–776. 10.1007/s10534-012-9523-0 22318507

[B153] Oexle H. Gnaiger E. Weiss G. (1999). Iron-dependent changes in cellular energy metabolism: influence on citric acid cycle and oxidative phosphorylation. *Biochim. Biophys. Acta* 1413 99–107. 10.1016/S0005-2728(99)00088-2 10556622

[B154] Okubo Y. Mikami Y. Kanemaru K. Iino M. (2018). Role of endoplasmic reticulum-mediated Ca(2+) signaling in neuronal cell death. *Antioxid. Redox Signal.* 29 1147–1157. 10.1089/ars.2018.7498 29361832

[B155] Orino K. Lehman L. Tsuji Y. Ayaki H. Torti S. V. Torti F. M. (2001). Ferritin and the response to oxidative stress. *Biochem. J.* 357 241–247. 10.1042/bj3570241 11415455 PMC1221947

[B156] Overk C. R. Rockenstein E. Florio J. Cheng Q. Masliah E. (2015). Differential calcium alterations in animal models of neurodegenerative disease: reversal by FK506. *Neuroscience* 310 549–560. 10.1016/j.neuroscience.2015.08.068 26341908 PMC4633337

[B157] Pantopoulos K. Hentze M. W. (1995). Rapid responses to oxidative stress mediated by iron regulatory protein. *EMBO J.* 14 2917–2924. 10.1002/j.1460-2075.1995.tb07291.x 7796817 PMC398411

[B158] Pantopoulos K. Weiss G. Hentze M. W. (1996). Nitric oxide and oxidative stress (H2O2) control mammalian iron metabolism by different pathways. *Mol. Cell. Biol.* 16 3781–3788. 10.1128/MCB.16.7.3781 8668195 PMC231374

[B159] Paradkar P. N. Roth J. A. (2006a). Nitric oxide transcriptionally down-regulates specific isoforms of divalent metal transporter (DMT1) via NF-kappaB. *J. Neurochem.* 96 1768–1777. 16539692 10.1111/j.1471-4159.2006.03702.x

[B160] Paradkar P. N. Roth J. A. (2006b). Post-translational and transcriptional regulation of DMT1 during P19 embryonic carcinoma cell differentiation by retinoic acid. *Biochem. J.* 394 173–183. 16232120 10.1042/BJ20051296 PMC1386015

[B161] Paradkar P. N. Zumbrennen K. B. Paw B. H. Ward D. M. Kaplan J. (2009). Regulation of mitochondrial iron import through differential turnover of mitoferrin 1 and mitoferrin 2. *Mol. Cell. Biol.* 29 1007–1016. 10.1128/MCB.01685-08 19075006 PMC2643804

[B162] Pchitskaya E. Popugaeva E. Bezprozvanny I. (2018). Calcium signaling and molecular mechanisms underlying neurodegenerative diseases. *Cell Calcium* 70 87–94. 10.1016/j.ceca.2017.06.008 28728834 PMC5748019

[B163] Pelizzoni I. Macco R. Morini M. F. Zacchetti D. Grohovaz F. Codazzi F. (2011). Iron handling in hippocampal neurons: activity-dependent iron entry and mitochondria-mediated neurotoxicity. *Aging Cell* 10 172–183. 10.1111/j.1474-9726.2010.00652.x 21108725

[B164] Pelizzoni I. Macco R. Zacchetti D. Grohovaz F. Codazzi F. (2008). Iron and calcium in the central nervous system: a close relationship in health and sickness. *Biochem. Soc. Trans.* 36 1309–1312. 10.1042/BST0361309 19021546

[B165] Pelizzoni I. Zacchetti D. Campanella A. Grohovaz F. Codazzi F. (2013). Iron uptake in quiescent and inflammation-activated astrocytes: a potentially neuroprotective control of iron burden. *Biochim. Biophys. Acta* 1832 1326–1333. 10.1016/j.bbadis.2013.04.007 23583428 PMC3787737

[B166] Pereira C. Ferreira C. Carvalho C. Oliveira C. (1996). Contribution of plasma membrane and endoplasmic reticulum Ca(2+)-ATPases to the synaptosomal [Ca2+]i increase during oxidative stress. *Brain Res.* 713 269–277. 10.1016/0006-8993(95)01554-X 8725000

[B167] Perier C. Tieu K. Guegan C. Caspersen C. Jackson-Lewis V. Carelli V. (2005). Complex I deficiency primes Bax-dependent neuronal apoptosis through mitochondrial oxidative damage. *Proc. Natl. Acad. Sci. U.S.A.* 102 19126–19131. 10.1073/pnas.0508215102 16365298 PMC1323177

[B168] Peters D. G. Connor J. R. Meadowcroft M. D. (2015). The relationship between iron dyshomeostasis and amyloidogenesis in Alzheimer’s disease: two sides of the same coin. *Neurobiol. Dis.* 81 49–65. 10.1016/j.nbd.2015.08.007 26303889 PMC4672943

[B169] Phillips J. D. Kinikini D. V. Yu Y. Guo B. Leibold E. A. (1996). Differential regulation of IRP1 and IRP2 by nitric oxide in rat hepatoma cells. *Blood* 87 2983–2992. 8639920

[B170] Piret J. P. Mottet D. Raes M. Michiels C. (2002). Is HIF-1alpha a pro- or an anti-apoptotic protein? *Biochem. Pharmacol.* 64 889–892. 12213583 10.1016/s0006-2952(02)01155-3

[B171] Ponka P. (2004). Hereditary causes of disturbed iron homeostasis in the central nervous system. *Ann. N. Y. Acad. Sci.* 1012 267–281. 10.1196/annals.1306.022 15105272

[B172] Popugaeva E. Pchitskaya E. Bezprozvanny I. (2018). Dysregulation of intracellular calcium signaling in alzheimer’s disease. *Antioxid. Redox Signal.* 29 1176–1188. 10.1089/ars.2018.7506 29890840 PMC6157344

[B173] Post M. R. Lieberman O. J. Mosharov E. V. (2018). Can interactions between alpha-synuclein, dopamine and calcium explain selective neurodegeneration in Parkinson’s disease? *Front. Neurosci.* 12:161. 10.3389/fnins.2018.00161 29593491 PMC5861202

[B174] Qian Z. M. Wu X. M. Fan M. Yang L. Du F. Yung W. H. (2011). Divalent metal transporter 1 is a hypoxia-inducible gene. *J. Cell Physiol.* 226 1596–1603. 10.1002/jcp.22485. 20945371

[B175] Ray P. D. Huang B. W. Tsuji Y. (2012). Reactive oxygen species (ROS) homeostasis and redox regulation in cellular signaling. *Cell. Signal.* 24 981–990. 10.1016/j.cellsig.2012.01.008 22286106 PMC3454471

[B176] Requejo-Aguilar R. Bolanos J. P. (2016). Mitochondrial control of cell bioenergetics in Parkinson’s disease. *Free Radic. Biol. Med.* 100 123–137. 10.1016/j.freeradbiomed.2016.04.012 27091692 PMC5065935

[B177] Riganti C. Doublier S. Viarisio D. Miraglia E. Pescarmona G. Ghigo D. (2009). Artemisinin induces doxorubicin resistance in human colon cancer cells via calcium-dependent activation of HIF-1alpha and P-glycoprotein overexpression. *Br. J. Pharmacol.* 156 1054–1066. 10.1111/j.1476-5381.2009.00117.x 19298255 PMC2697684

[B178] Rimessi A. Bonora M. Marchi S. Patergnani S. Marobbio C. M. Lasorsa F. M. (2013). Perturbed mitochondrial Ca2+ signals as causes or consequences of mitophagy induction. *Autophagy* 9 1677–1686. 10.4161/auto.24795 24121707

[B179] Riquelme D. Alvarez A. Leal N. Adasme T. Espinoza I. Valdes J. A. (2011). High-frequency field stimulation of primary neurons enhances ryanodine receptor-mediated Ca2+ release and generates hydrogen peroxide, which jointly stimulate NF-kappaB activity. *Antioxid. Redox Signal.* 14 1245–1259. 10.1089/ars.2010.3238 20836702

[B180] Rosales-Corral S. Acuna-Castroviejo D. Tan D. X. Lopez-Armas G. Cruz-Ramos J. Muñoz R. (2012). Accumulation of exogenous amyloid-beta peptide in hippocampal mitochondria causes their dysfunction: a protective role for melatonin. *Oxid. Med. Cell. Longev.* 2012:843649. 10.1155/2012/843649 22666521 PMC3359765

[B181] Rosi S. Ramirez-Amaya V. Hauss-Wegrzyniak B. Wenk G. L. (2004). Chronic brain inflammation leads to a decline in hippocampal NMDA-R1 receptors. *J Neuroinflammation* 1:12. 10.1186/1742-2094-1-12 15285803 PMC500869

[B182] Rothwell N. J. Luheshi G. N. (2000). Interleukin 1 in the brain: biology, pathology and therapeutic target. *Trends Neurosci.* 23 618–625. 10.1016/S0166-2236(00)01661-1 11137152

[B183] Rousseau E. Michel P. P. Hirsch E. C. (2013). The iron-binding protein lactoferrin protects vulnerable dopamine neurons from degeneration by preserving mitochondrial calcium homeostasis. *Mol. Pharmacol.* 84 888–898. 10.1124/mol.113.087965 24077968

[B184] Ruiz A. Alberdi E. Matute C. (2018). Mitochondrial division inhibitor 1 (mdivi-1) protects neurons against excitotoxicity through the modulation of mitochondrial function and intracellular Ca(2+) signaling. *Front. Mol. Neurosci.* 11:3. 10.3389/fnmol.2018.00003 29386996 PMC5776080

[B185] Sanmartin C. D. Paula-Lima A. C. Garcia A. Barattini P. Hartel S. Núñez M. T. (2014). Ryanodine receptor-mediated Ca(2+) release underlies iron-induced mitochondrial fission and stimulates mitochondrial Ca(2+) uptake in primary hippocampal neurons. *Front. Mol. Neurosci.* 7:13. 10.3389/fnmol.2014.00013 24653672 PMC3949220

[B186] Schiavone S. Sorce S. Dubois-Dauphin M. Jaquet V. Colaianna M. Zotti M. (2009). Involvement of NOX2 in the development of behavioral and pathologic alterations in isolated rats. *Biol. Psychiatry* 66 384–392. 10.1016/j.biopsych.2009.04.033 19559404

[B187] Sen C. K. (2001). Antioxidants in exercise nutrition. *Sports Med.* 31 891–908. 10.2165/00007256-200131130-00001 11708399

[B188] Sherer T. B. Betarbet R. Testa C. M. Seo B. B. Richardson J. R. Kim J. H. (2003). Mechanism of toxicity in rotenone models of Parkinson’s disease. *J. Neurosci.* 23 10756–10764. 10.1523/JNEUROSCI.23-34-10756.2003 14645467 PMC6740985

[B189] Shih R. H. Wang C. Y. Yang C. M. (2015). NF-kappaB signaling pathways in neurological inflammation: a mini review. *Front. Mol. Neurosci.* 8:77. 10.3389/fnmol.2015.00077 26733801 PMC4683208

[B190] Shimohama S. Tanino H. Kawakami N. Okamura N. Kodama H. Yamaguchi T. (2000). Activation of NADPH oxidase in Alzheimer’s disease brains. *Biochem. Biophys. Res. Commun.* 273 5–9. 10.1006/bbrc.2000.2897 10873554

[B191] Siddiq A. Ayoub I. A. Chavez J. C. Aminova L. Shah S. Lamanna J. C. (2005). Hypoxia-inducible factor prolyl 4-hydroxylase inhibition. A target for neuroprotection in the central nervous system. *J. Biol. Chem.* 280 41732–41743. 10.1074/jbc.M504963200 16227210 PMC2586128

[B192] Siegert I. Schodel J. Nairz M. Schatz V. Dettmer K. Dick C. (2015). Ferritin-Mediated iron sequestration stabilizes hypoxia-inducible factor-1alpha upon LPS activation in the presence of ample oxygen. *Cell Rep.* 13 2048–2055. 10.1016/j.celrep.2015.11.005 26628374

[B193] Siklos L. Engelhardt J. I. Alexianu M. E. Gurney M. E. Siddique T. Appel S. H. (1998). Intracellular calcium parallels motoneuron degeneration in SOD-1 mutant mice. *J. Neuropathol. Exp. Neurol.* 57 571–587. 10.1097/00005072-199806000-00005 9630237

[B194] Sirabella R. Valsecchi V. Anzilotti S. Cuomo O. Vinciguerra A. Cepparulo P. (2018). Ionic homeostasis maintenance in ALS: focus on new therapeutic targets. *Front. Neurosci.* 12:510. 10.3389/fnins.2018.00510 30131665 PMC6090999

[B195] Smith T. G. Robbins P. A. Ratcliffe P. J. (2008). The human side of hypoxia-inducible factor. *Br. J. Haematol.* 141 325–334. 10.1111/j.1365-2141.2008.07029.x 18410568 PMC2408651

[B196] Sorce S. Krause K. H. (2009). NOX enzymes in the central nervous system: from signaling to disease. *Antioxid. Redox Signal.* 11 2481–2504. 10.1089/ARS.2009.2578 19309263

[B197] Sorce S. Stocker R. Seredenina T. Holmdahl R. Aguzzi A. Chio A. (2017). NADPH oxidases as drug targets and biomarkers in neurodegenerative diseases: what is the evidence? *Free Radic. Biol. Med.* 112 387–396. 10.1016/j.freeradbiomed.2017.08.006 28811143

[B198] Soum E. Drapier J. C. (2003). Nitric oxide and peroxynitrite promote complete disruption of the [4Fe-4S] cluster of recombinant human iron regulatory protein 1. *J. Biol. Inorg. Chem.* 8 226–232. 10.1007/s00775-002-0412-9 12459918

[B199] Stefani I. C. Wright D. Polizzi K. M. Kontoravdi C. (2012). The role of ER stress-induced apoptosis in neurodegeneration. *Curr. Alzheimers Res.* 9 373–387. 10.2174/156720512800107618 22299619

[B200] Stockwell B. R. Friedmann Angeli J. P. Bayir H. Bush A. I. Conrad M. Dixon S. J. (2017). Ferroptosis: a regulated cell death nexus linking metabolism, redox biology, and disease. *Cell* 171 273–285. 10.1016/j.cell.2017.09.021 28985560 PMC5685180

[B201] Strehler E. E. Thayer S. A. (2018). Evidence for a role of plasma membrane calcium pumps in neurodegenerative disease: recent developments. *Neurosci. Lett.* 663 39–47. 10.1016/j.neulet.2017.08.035 28827127 PMC5816698

[B202] Surace M. J. Block M. L. (2012). Targeting microglia-mediated neurotoxicity: the potential of NOX2 inhibitors. *Cell. Mol. Life Sci.* 69 2409–2427. 10.1007/s00018-012-1015-4 22581365 PMC3677079

[B203] Sureda A. Hebling U. Pons A. Mueller S. (2005). Extracellular H2O2 and not superoxide determines the compartment-specific activation of transferrin receptor by iron regulatory protein 1. *Free Radic. Res.* 39 817–824. 10.1080/10715760500164045 16036361

[B204] Surmeier D. J. Guzman J. N. Sanchez-Padilla J. (2010). Calcium, cellular aging, and selective neuronal vulnerability in Parkinson’s disease. *Cell. Calcium* 47 175–182. 10.1016/j.ceca.2009.12.003 20053445 PMC3235732

[B205] Surmeier D. J. Guzman J. N. Sanchez-Padilla J. Goldberg J. A. (2011). The origins of oxidant stress in Parkinson’s disease and therapeutic strategies. *Antioxid. Redox Signal.* 14 1289–1301. 10.1089/ars.2010.3521 20712409 PMC3048813

[B206] Swaiman K. F. (1991). Hallervorden-Spatz syndrome and brain iron metabolism. *Arch. Neurol.* 48 1285–1293. 10.1001/archneur.1991.00530240091029 1845035

[B207] Tan S. Sagara Y. Liu Y. Maher P. Schubert D. (1998). The regulation of reactive oxygen species production during programmed cell death. *J. Cell. Biol.* 141 1423–1432. 10.1083/jcb.141.6.1423 9628898 PMC2132785

[B208] Tangudu N. K. Alan B. Vinchi F. Worle K. Lai D. Vettorazzi S. (2018). Scavenging reactive oxygen species production normalizes ferroportin expression and ameliorates cellular and systemic iron disbalances in hemolytic mouse model. *Antioxid. Redox Signal.* 29 484–499. 10.1089/ars.2017.7089 29212341 PMC6034398

[B209] Thomas G. M. Huganir R. L. (2004). MAPK cascade signalling and synaptic plasticity. *Nat. Rev. Neurosci.* 5 173–183. 10.1038/nrn1346 14976517

[B210] Tonelli C. Chio I. I. C. Tuveson D. A. (2018). Transcriptional regulation by Nrf2. *Antioxid. Redox Signal.* 29 1727–1745. 10.1089/ars.2017.7342 28899199 PMC6208165

[B211] Tong W. H. Rouault T. A. (2007). Metabolic regulation of citrate and iron by aconitases: role of iron-sulfur cluster biogenesis. *Biometals* 20 549–564. 10.1007/s10534-006-9047-6 17205209

[B212] Tonnies E. Trushina E. (2017). Oxidative stress, synaptic dysfunction, and Alzheimer’s Disease. *J. Alzheimers Dis.* 57 1105–1121. 10.3233/JAD-161088 28059794 PMC5409043

[B213] Urrutia P. Aguirre P. Esparza A. Tapia V. Mena N. P. Arredondo M. (2013). Inflammation alters the expression of DMT1, FPN1 and hepcidin, and it causes iron accumulation in central nervous system cells. *J. Neurochem.* 126 541–549. 10.1111/jnc.12244 23506423

[B214] Urrutia P. J. Mena N. P. Núñez M. T. (2014). The interplay between iron accumulation, mitochondrial dysfunction, and inflammation during the execution step of neurodegenerative disorders. *Front. Pharmacol.* 5:38. 10.3389/fphar.2014.00038 24653700 PMC3948003

[B215] Vila M. Przedborski S. (2003). Targeting programmed cell death in neurodegenerative diseases. *Nat. Rev. Neurosci.* 4 365–375. 10.1038/nrn1100 12728264

[B216] Wang J. Song N. Jiang H. Wang J. Xie J. (2013). Pro-inflammatory cytokines modulate iron regulatory protein 1 expression and iron transportation through reactive oxygen/nitrogen species production in ventral mesencephalic neurons. *Biochim. Biophys. Acta* 1832 618–625. 10.1016/j.bbadis.2013.01.021 23376588

[B217] Wang Q. M. Xu Y. Y. Liu S. Ma Z. G. (2017). Isradipine attenuates MPTP-induced dopamine neuron degeneration by inhibiting up-regulation of L-type calcium channels and iron accumulation in the substantia nigra of mice. *Oncotarget* 8 47284–47295. 10.18632/oncotarget.17618 28521299 PMC5564564

[B218] Wang Y. Yang J. Yi J. (2012). Redox sensing by proteins: oxidative modifications on cysteines and the consequent events. *Antioxid. Redox Signal.* 16 649–657. 10.1089/ars.2011.4313 21967570

[B219] Ward R. J. Zucca F. A. Duyn J. H. Crichton R. R. Zecca L. (2014). The role of iron in brain ageing and neurodegenerative disorders. *Lancet Neurol.* 13 1045–1060. 10.1016/S1474-4422(14)70117-6 25231526 PMC5672917

[B220] Wardrop S. L. Watts R. N. Richardson D. R. (2000). Nitrogen monoxide activates iron regulatory protein 1 RNA-binding activity by two possible mechanisms: effect on the [4Fe-4S] cluster and iron mobilization from cells. *Biochemistry* 39 2748–2758. 10.1021/bi991099t 10704227

[B221] Weinreb O. Amit T. Bar-Am O. Youdim M. B. (2016). Neuroprotective effects of multifaceted hybrid agents targeting MAO, cholinesterase, iron and beta-amyloid in ageing and Alzheimer’s disease. *Br. J. Pharmacol.* 173 2080–2094. 10.1111/bph.13318 26332830 PMC4908201

[B222] Wiethoff S. Houlden H. (2017). Neurodegeneration with brain iron accumulation. *Handb. Clin. Neurol.* 145 157–166. 10.1016/B978-0-12-802395-2.00011-0 28987166

[B223] Wilkinson N. Pantopoulos K. (2014). The IRP/IRE system in vivo: insights from mouse models. *Front. Pharmacol.* 5:176. 10.3389/fphar.2014.00176 25120486 PMC4112806

[B224] Wu D. C. Jackson-Lewis V. Vila M. Tieu K. Teismann P. Vadseth C. (2002). Blockade of microglial activation is neuroprotective in the 1-methyl-4-phenyl-1,2,3,6-tetrahydropyridine mouse model of Parkinson disease. *J. Neurosci.* 22 1763–1771. 10.1523/JNEUROSCI.22-05-01763.2002 11880505 PMC6758858

[B225] Wu G. Fang Y. Z. Yang S. Lupton J. R. Turner N. D. (2004). Glutathione metabolism and its implications for health. *J. Nutr.* 134 489–492. 10.1093/jn/134.3.489 14988435

[B226] Wu G. Y. Deisseroth K. Tsien R. W. (2001). Activity-dependent CREB phosphorylation: convergence of a fast, sensitive calmodulin kinase pathway and a slow, less sensitive mitogen-activated protein kinase pathway. *Proc. Natl. Acad. Sci. U.S.A.* 98 2808–2813. 10.1073/pnas.051634198 11226322 PMC30221

[B227] Xiao J. Lv Y. Lin B. Tipoe G. L. Youdim M. B. Xing F. (2015). A novel antioxidant multitarget iron chelator M30 protects hepatocytes against ethanol-induced injury. *Oxid. Med. Cell. Longev.* 2015:607271. 10.1155/2015/607271 25722794 PMC4334871

[B228] Yang W. S. Sriramaratnam R. Welsch M. E. Shimada K. Skouta R. Viswanathan V. S. (2014). Regulation of ferroptotic cancer cell death by GPX4. *Cell* 156 317–331. 10.1016/j.cell.2013.12.010 24439385 PMC4076414

[B229] Yeh T. L. Leissing T. M. Abboud M. I. Thinnes C. C. Atasoylu O. Holt-Martyn J. P. (2017). Molecular and cellular mechanisms of HIF prolyl hydroxylase inhibitors in clinical trials. *Chem. Sci.* 8 7651–7668. 10.1039/c7sc02103h 29435217 PMC5802278

[B230] Yermolaieva O. Brot N. Weissbach H. Heinemann S. H. Hoshi T. (2000). Reactive oxygen species and nitric oxide mediate plasticity of neuronal calcium signaling. *Proc. Natl. Acad. Sci. U.S.A.* 97 448–453. 10.1073/pnas.97.1.448 10618438 PMC26683

[B231] Yuan G. Nanduri J. Bhasker C. R. Semenza G. L. Prabhakar N. R. (2005). Ca2+/calmodulin kinase-dependent activation of hypoxia inducible factor 1 transcriptional activity in cells subjected to intermittent hypoxia. *J. Biol. Chem.* 280 4321–4328. 10.1074/jbc.M407706200 15569687

[B232] Yuan G. Nanduri J. Khan S. Semenza G. L. Prabhakar N. R. (2008). Induction of HIF-1alpha expression by intermittent hypoxia: involvement of NADPH oxidase, Ca2+ signaling, prolyl hydroxylases, and mTOR. *J. Cell. Physiol.* 217 674–685. 10.1002/jcp.21537 18651560 PMC2696817

[B233] Zaidi A. (2010). Plasma membrane Ca-ATPases: targets of oxidative stress in brain aging and neurodegeneration. *World J. Biol. Chem.* 1 271–280. 10.4331/wjbc.v1.i9.271 21537484 PMC3083975

[B234] Zaidi A. Michaelis M. L. (1999). Effects of reactive oxygen species on brain synaptic plasma membrane Ca(2+)-ATPase. *Free Radic. Biol. Med.* 27 810–821. 10.1016/S0891-5849(99)00128-8 10515585

[B235] Zecca L. Youdim M. B. Riederer P. Connor J. R. Crichton R. R. (2004). Iron, brain ageing and neurodegenerative disorders. *Nat. Rev. Neurosci.* 5 863–873. 10.1038/nrn1537 15496864

[B236] Zhang W. Yan Z. F. Gao J. H. Sun L. Huang X. Y. Liu Z. (2014). Role and mechanism of microglial activation in iron-induced selective and progressive dopaminergic neurodegeneration. *Mol. Neurobiol.* 49 1153–1165. 10.1007/s12035-013-8586-4 24277523 PMC4878835

[B237] Zhang X. Zhou K. Wang R. Cui J. Lipton S. A. Liao F. F. (2007). Hypoxia-inducible factor 1alpha (HIF-1alpha)-mediated hypoxia increases BACE1 expression and beta-amyloid generation. *J. Biol. Chem.* 282 10873–10880. 10.1074/jbc.M608856200 17303576

[B238] Zhang Y. Kong W. N. Chai X. Q. (2018). Compound of icariin, astragalus, and puerarin mitigates iron overload in the cerebral cortex of Alzheimer’s disease mice. *Neural Regen. Res.* 13 731–736. 10.4103/1673-5374.230302 29722328 PMC5950686

[B239] Zorov D. B. Isaev N. K. Plotnikov E. Y. Zorova L. D. Stelmashook E. V. Vasileva A. K. (2007). The mitochondrion as janus bifrons. *Biochemistry* 72 1115–1126. 18021069 10.1134/s0006297907100094

